# Tumor Environment Regression Therapy Implemented by Switchable Prune‐to‐Essence Nanoplatform Unleashed Systemic Immune Responses

**DOI:** 10.1002/advs.202303715

**Published:** 2023-10-24

**Authors:** Xianzhou Huang, Lu Li, Chunqing Ou, Meiling Shen, Xinchao Li, Miaomiao Zhang, Rui Wu, Xiaorong Kou, Ling Gao, Furong Liu, Rui Luo, Qinjie Wu, Changyang Gong

**Affiliations:** ^1^ Department of Biotherapy Cancer center and State Key Laboratory of Biotherapy West China Hospital Sichuan University Chengdu 610041 China; ^2^ Department of Medical Oncology Cancer Center West China Hospital Sichuan University Chengdu 610041 China

**Keywords:** antiangiogenesis, cancer associated fibroblasts, immune memory, systemic immune response, tumor environment regression therapy

## Abstract

Coevolution of tumor cells and surrounding stroma results in protective protumoral environment, in which abundant vessel, stiff structure and immunosuppression promote each other, cooperatively incurring deterioration and treatment compromise. Reversing suchenvironment may transform tumors from treatment‐resistant to treatment‐vulnerable. However, effective reversion requires synergistic comprehensive regression of such environment under precise control. Here, the first attempt to collaboratively retrograde coevolutionary tumor environment to pre‐oncogenesis status, defined as tumor environment regression therapy, is made for vigorous immune response eruption by a switchable prune‐to‐essence nanoplatform (Pres) with simplified composition and fabrication process. Through magnetic targeting and multimodal imaging of Pres, tumor environment regression therapy is guided, optimized and accomplished in a trinity way: Antiangiogenesis is executed to rarefy vessels to impede tumor progression. By seizing the time, cancer associated fibroblasts are eliminated to diminish collagen and loosen the stiff structure for deep penetration of Pres, which alternately functioned in deeper tumors, forming a positive feedback loop. Through this loop, immune cell infiltration, immunosuppression mitigation and immunogenic cells death induction are all fulfilled and further escalated in the regressed environment. These transformations consequently unleashed systemic immune responses and generated immune memory against carcinoma. This study provides new insights intotreatment of solid tumors.

## Graphical abstract

A strategy named tumor environment regression therapy is proposed to regress the coevolutionary tumor environment into the status before tumorigenesis coevolution for antitumor immunity enhancement by switchable Pres, which turns tumor environment from “indestructible fortress” into “vulnerable village” and unleashes vigorous systemic immune responses against TNBC. This strategy consequently accomplishes long‐term and effective inhibition of TNBC.

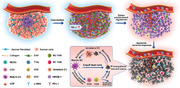

## 1 Introduction

Tumor environment is a dynamic coevolution product of tumor cells and corresponding stromata.^[^ ^1^, ^2^, ^3^, ^4^, ^5^ ^]^ This reciprocal interaction results in high microvascular density, cancer associated fibroblast (CAF) activation and abundant desmoplasia to form and support the fibrosis environment, as an “indestructible fortress”, in malignant solid tumors, such as triple negative breast cancer (TNBC).^[^ ^5^, ^6^, ^7^, ^8^, ^9^, ^10^ ^]^ While new vessels foster and accelerate tumor progression and aggression, CAFs build up dense interstitial collagens in tumor environment to block the infiltration of drugs and lymphocytes.^[^ ^11^, ^12^, ^13^, ^14^, ^15^, ^16^, ^17^, ^18^, ^19^ ^]^ Additionally, such a stifling tumor environment compromises treatments by reinforcing immunosuppression to mute immune responses.^[15^ ^]^ The absence of effective immune cells accompanied with immunosuppression in tumor environment contributes to the low responsiveness of tumor cells‐targeted, lymphocyte‐targeted or immune‐checkpoint‐targeted immunotherapies.^[^ ^19^, ^20^, ^21^ ^]^

Accordingly, therapies that regress the stiffening vascular‐dense immunosuppressive environment into the status before tumorigenesis coevolution, defined as tumor environment regression therapy here, may elicit an effective systemic defensive response. While neovascularization repression can rarefy tumoral vessels and impede the further growth of carcinomas in avascular regions to buy additional time for therapies,^[^ ^22^, ^23^ ^]^ CAFs elimination will loosen the stiff structure to facilitate drug and lymphocyte infiltration, and to relieve immunosuppression from self‐tolerance, subject to which malignant coevolutionary oncogenesis occurs.^[^ ^24^, ^25^, ^26^ ^]^ Together, tumor environment regression will be achieved to transform “indestructible fortress” into “vulnerable village”, to recruit and activate antigen presenting cells (APCs) to escalate effective immune responses through instigation of immunogenic cell death (ICD) aroused by stresses like heat,^[^ ^27^, ^28^, ^29^, ^30^, ^31^, ^32^, ^33^, ^34^, ^35^ ^]^ and to win the anti‐carcinoma war via improved penetration depth of agents generating these lethal stresses.

To fulfill such tumor environment regression therapy, rational design to achieve sophisticated integration of antiangiogenesis, tumor microenvironment remodeling, immunomodulation and other approaches is inevitable. We here made these multifaceted functions true and consolidation as the first attempt by a switchable prune‐to‐essence (Pres) nanoplatform obtained from the simple introduction of sunitinib (Su) and superparamagnetic Fe_3_O_4_ nanoparticles into polydopamine (PDA). Su could not only repress neovascularization in tumor environment, but also stimulate immune responses by inhibiting the activity of immune suppressive cells.^[^ ^23^, ^26^, ^32^, ^33^ ^]^ While superparamagnetic Fe_3_O_4_ nanoparticles could equip our Pres with the capability of magnetic targeting, PDA could subsequently induce effective photothermal effect specifically in tumor tissues to eliminate CAFs and instigate ICD of tumor cells.^[^ ^29^, ^30^, ^31^, ^34^, ^36^ ^]^ Furthermore, with the guidance of superparamagnetism for convenient targeting and multimodal imaging endowed from both superparamagnetic Fe_3_O_4_ nanoparticles and PDA for irradiation timing optimization,^[^ ^37^, ^38^, ^39^, ^40^, ^41^, ^42^ ^]^ switchable Pres regressed tumor environment in a trinity way: Antiangiogenesis was executed to rarely tumor‐supportive vessels to impede tumor progression. During the saved time, Pres eliminated CAFs to diminish collagen and loosen tumor structure for penetration depth improvement. The improved penetration depth of Pres and tumor environment regression worked as a positive feedback loop. Through this loop, Pres further removed immunosuppression and improved immune cells recruitment deep into the retrograded tumor environment.^[^ ^43^, ^44^, ^45^ ^]^ Consequently, instigated by ICD of tumor cells, systemic immune responses were unleashed and strengthened, and immune memory was generated to curb tumor progression. Our study provides new insights into the immunotherapy of solid tumors.

## 2 Results

### 2.1 Pres Exhibited Preferable Stability and Switchable Drug Release Behavior

**Figure 1:**
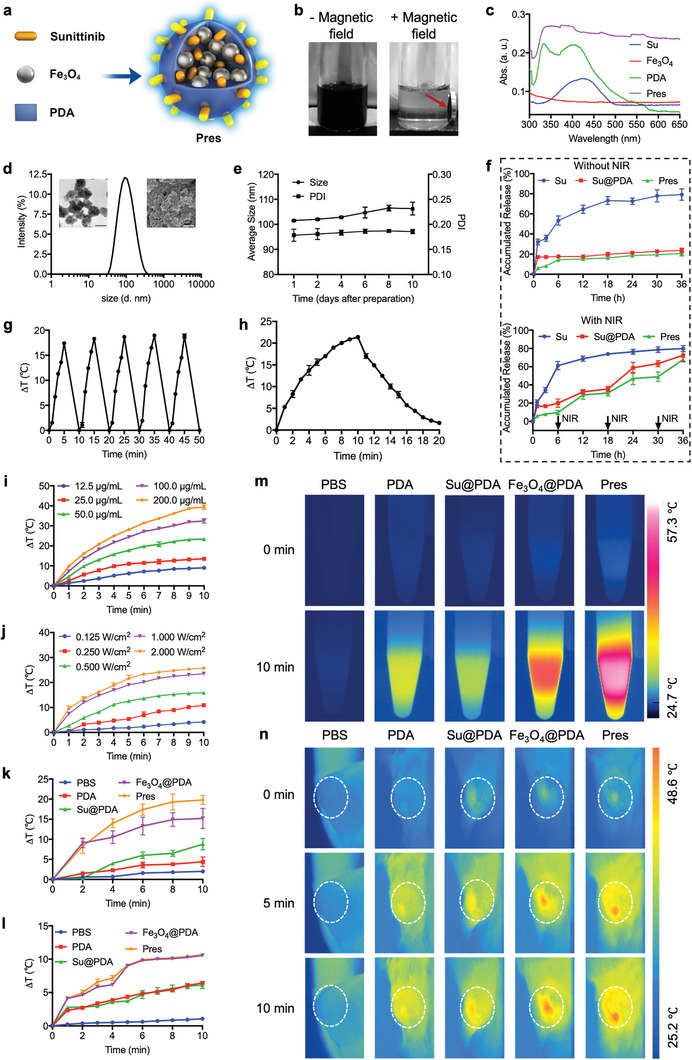
Pres with preferable stability and photothermal properties was switchable. a) Illustration of Pres obtained by simply introducing Su and superparamagnetic Fe_3_O_4_ nanoparticles into PDA. b) Pres solution was black in appearance without magnetic field, and turned to transparent after Pres was attracted (red arrow) by magnetic field. c) Scanning spectra of Pres, Su, PDA and superparamagnetic Fe_3_O_4_ nanoparticles. d) Size distribution of Pres. Transmission electron microscope (TEM) image (left, scale bar, 100 nm) and scanning electron microscope (SEM) image (right, scale bar, 500 nm) were inserted. e) Average size and PDI of Pres dispersed in PBS were monitored for 10 days. f) Drug release behaviors of Su from free Su, Su@PDA and Pres without or with 808 nm irradiation (black arrows) in 36 h, respectively (n = 3). Stimulated release of Su post irradiation was determined in the next time interval. g) On‐off temperature curve of Pres (100 µg mL^−1^) under 808 nm irradiation (1.0 W cm^−2^). h) Temperature change of Pres during heat‐cooling period with 10 min of irradiation. i,j) Temperature changes of Pres solution under irradiation for 10 min with different concentrations or laser powers, respectively (n = 3). Photothermal temperature variations k,l) and corresponding thermal images m,n) of Pres, PBS, PDA, Su@PDA and Fe_3_O_4_@PDA under irradiation were monitored in vitro k,m) and in vivo l,n) (n = 3). All data are presented as mean ± SD.

Pres (**Figure** **1**a) was obtained by simply introducing Su and Fe_3_O_4_ nanoparticles into the preparation process of PDA under generous stirring with ultrasonic. After separation and collection, the obtained Pres solution was black in appearance, exhibiting the color of PDA as shown in Figure 1b. Pres could be easily attracted and purified with the help of magnetic field, suggesting that the recruitment of superparamagnetic Fe_3_O_4_ nanoparticles successfully endowed Pres with magnetic targeting capability. The results of UV–vis‐NIR spectrum (Figure 1c) and detection of each constituent further proved the successful preparation of Pres. The composition ratios of PDA, Fe_3_O_4_ and Su in Pres were determined to be ≈73.5%, ≈25.0% and ≈1.5% (wt.%), respectively.

The obtained Pres formed a nearly spherical structure with diameter of 102 ± 3 nm (Figure 1d). Meanwhile, Pres was evidenced to be well‐dispersed and stable for at least 10 days in phosphate buffer saline (PBS) (Figure 1e; Figure S1, Supporting Information) and 24 h in 10% fetal bovine serum (FBS) (Figure S2, Supporting Information). This stability in structure had some influence on the drug release behavior of Pres. Only 20.74 ± 2.54% Su was released in 36 h without irradiation, far less than that of free Su (Figure 1f). However, by executing 808 nm laser irradiation, the release of Su in Pres could be switchable. As shown in Figure 1f, with irradiation, the switch turned “on”. Much higher levels of Su were detected. Without irradiation, the switch turned “off”. Much less Su was released. After 3 irradiations, 67.87 ± 2.54% Su was released from Pres (Figure 1f), fully sufficient for in vivo treatment. Furthermore, the switchable release of Su and the magnetic targeting capability of Pres would reduce the toxicity of Su to untargeted tissues and improve Su accumulation in targeted tumors.

### 2.2 Pres exhibited Favorable Photothermal Properties

Photothermal stability of the switchable Pres was evidenced with at least 5 “on‐off” cycles (Figure 1g). Additionally, photothermal conversion efficiency of Pres was calculated to be 38.3% according to the heat‐cooling period (Figure 1h) and corresponding τ_s_ (Figure S3, Supporting Information). Such calculation was performed at 1.0 W cm^−2^ 808 nm laser with 100 µg mL^−1^ Pres, which was previously determined according to the preferred temperature changes in 10 min under 808 nm irradiation with different Pres concentrations (Figure 1i) or different laser powers (Figure 1j). Furthermore, photothermal property of Pres was evaluated in vitro (Figure 1k,m) and in vivo (Figure 1l,n). The highest temperature increase (up to ≈57.3 °C) was observed in Pres solution in 10 min in vitro. And both Pres and Fe_3_O_4_@PDA exhibited significantly, and similarly higher temperature increases than other groups after 5 min (reaching ≈47.2 °C) in vivo, indicating that they generated equivalent heat in tumor sites after irradiation for 5 min. To control variates and to better evaluate other effects (such as antiangiogenesis) in vivo, the following experiments were conducted with irradiation for 5 min to induce photothermal effects, if necessary.

### 2.3 Pres Repressed Neovascularization

**Figure 2:**
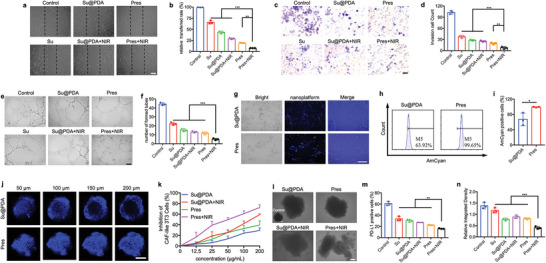
Switchable Pres repressed neovascularization and eliminated CAFs to loosen tumor structure and relieve immunosuppression from PD‐L1 in vitro. a,b) Images and quantitative analysis of wound healing of HUVEC cells. Dotted lines represented the original wound margins. Scale bar, 400 µm. c,d) Images and quantitative analysis of invasion assay of HUVEC cells. Scale bar, 200 µm. e,f) Images and quantitative analysis of tube formation of HUVEC cells. Scale bar, 400 µm. g) Fluorescence microscope images of cellular uptake analysis on CAF‐like 3T3 cells. Scale bar, 100 µm. h) Cellular uptake in CAF‐like 3T3 cells detected by FCM. i) Quantitative analysis of cellular uptake. j) CLSM images of 4T1 and CAF‐like 3T3 hybrid spheroids at 50, 100, 150, and 200 µm depth sections. Blue signals represented the PDA components in each nanoplatform. k) MTT assay on CAF‐like 3T3 cells. l) Images of 4T1 and CAF‐like 3T3 hybrid spheroids after different treatments. The disassembly of the hybrid spheroid treated with Pres and irradiation indicated loosened structure of hybrid spheroid. Scale bar, 100 µm. m) PD‐L1 positive 4T1 cells detected by FCM. Analyzed 4T1 cells were induced by CAF‐like 3T3 cells with different pre‐treatments. n) Statistical analysis of relative integrated density calculated via ratios of the gray value of PD‐L1 to that of GAPDH in each group in Supporting Figure 21. Statistical significance was determined by one‐way ANOVA with Tukey's HSD multiple comparisons post‐hoc test. All data are presented as mean ± SD, *n* = 3, * *p* < 0.05, ** *p* < 0.01, *** *p* < 0.001.

The ability of Pres in impeding the further growth of carcinomas in avascular regions to save time for therapy was next evaluated by antiangiogenesis efficiency. Results showed that, although Su‐containing groups displayed much stronger antiangiogenesis effects than non‐Su groups, unlocked Pres exhibited the highest antiangiogenesis efficiency of all (Figures S4–S9, Supporting Information). Specifically, unlocked Pres significantly restricted the wound healing of HUVEC cells in that the fewest cells were observed within the original wound (**Figure** **2**a,b; Figures S4 and S5, Supporting Information). Meanwhile, unlocked Pres observably hindered the invasion of HUVEC cells (Figure 2c; Figure S6, Supporting Information) in that remarkably fewer cells were counted when treated with unlocked Pres (Figure 2d; Figure S7, Supporting Information). Effect of switchable Pres on tube formation was further explored. Results showed that HUVEC cells formed obviously less tubes (Figure 2e,f; Figures S8 and S9, Supporting Information). All these findings suggested that Pres exhibited excellent switchable antiangiogenesis effect which could efficiently rarefy tumoral vessels and slow the growth of tumor to buy additional time for therapy.

### 2.4 Pres Erased CAFs for Deep Tumor Penetration and Immunosuppression Relief from PD‐L1

As the main component of stromal cells, CAFs promote tumor progression.^[15^ ^]^ NIH 3T3 cells could be activated by transforming growth factor‐β (TGF‐β) into CAF‐like phenotype, characterized with obviously upregulated α‐smooth muscle actin (α‐SMA) expression (Figure S10, Supporting Information). In addition, Pres exhibited fluorescence at ≈464 nm, where these signals of PDA component became much stronger after degradation (Figure S11, Supporting Information), which enabled the detection of Pres by flow cytometry (FCM) and confocal laser scanning microscopy (CLSM). According to the results in Figure 2g–i and Figures S12–S14 (Supporting Information), CAFs tended to endocytose Pres, which endowed Pres with deeper penetration depth in 4T1 and CAF‐like 3T3 hybrid spheroids (Figure 2j; Figure S15, Supporting Information). Furthermore, with improved penetration depth, deeper cells could be annihilated by Pres. Significantly stronger cytotoxicity or damage was observed in Pres+NIR group in 3‐(4,5‐dimethylthiazol‐2‐yl)−2,5‐diphenyltetrazolium bromide (MTT) assay of CAF‐like 3T3 cells (Figure 2k; Figure S16, Supporting Information) or 4T1 and CAF‐like 3T3 cells hybrid spheroid destruction analysis (Figure 2l; Figure S17, Supporting Information). The findings evidenced that switchable Pres could dive deeper to loosen tumor structure, which might lead to the infiltration of more Pres and immune cells.

Additionally, the influence of switchable Pres on programmed death ligand‐1 (PD‐L1) expression of tumor cells, which was reported^[^ ^46^, ^47^, ^48^ ^]^ and proved able to be induced and promoted by CAFs (Figures S18 and S19, Supporting Information), was explored. In brief, PD‐L1 expression in 4T1 cells after induction by CAF‐like 3T3 cells with different pre‐treatments was detected. Results showed that unlocked Pres significantly reduced the effect of CAF‐like 3T3 cells on PD‐L1 expression of 4T1 cells, since the least PD‐L1 positive 4T1 cells were counted in Figure 2m and Figure S20 (Supporting Information), and the smallest relative integrated density of PD‐L1 to glyceraldehyde‐3‐phosphate dehydrogenase (GAPDH) was detected in Pres+NIR group in Figure 2n and Figures S21 and S22 (Supporting Information). These findings verified that switchable Pres could remarkably reduce PD‐L1 expression in 4T1 cells through eliminating the induction effect of CAFs.

### 2.5 Switchable Pres Instigated Effective ICD of 4T1 Cells

**Figure 3:**
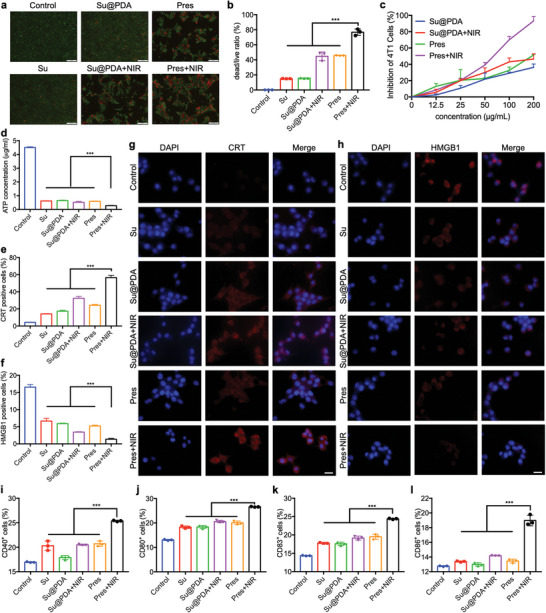
Switchable Pres induced ICD of 4T1 cells and activated BMDCs. a) Live‐dead staining assay of 4T1 cells with different treatments detected by fluorescence microscope. Scale bar, 200 µm. b) Live/dead ratios of 4T1 cells with different treatments detected by FCM. c) MTT assay on 4T1 cells with different treatments. d) ATP concentrations in 4T1 cells with different treatments. e,f) FCM analysis of CRT e) and HMGB1 f) levels in 4T1 cells with different treatments. g,h) CLSM images of CRT exposure (red, g) and HMGB1 residue (red, h) in 4T1 cells with different treatments. Cell nuclei were stained with DAPI (blue). Scale bar, 100 µm. i–l) CD11c^+^CD40^+^ i), CD11c^+^CD80^+^ j), CD11c^+^CD83^+^ k), CD11c^+^CD86^+^ l) BMDCs induced by 4T1 cells with different pre‐treatments detected by FCM. Statistical significance was determined by one‐way ANOVA with Tukey's HSD multiple comparisons post‐hoc test. All data are presented as mean ± SD, *n* = 3, ** *p* < 0.01, *** *p* < 0.001.

Both Pres and Su@PDA could be efficiently cellular uptake by 4T1 cells (Figures S23–S25, Supporting Information). Meanwhile, switchable Pres induced the highest proportion of 4T1 cell death, since obviously more dead cells (red) were observed (**Figure** **3**a; Figure S26, Supporting Information) and the dead/live cell ratio was the highest of all in Pres+NIR group (Figure 3b; Figure S27). This cytotoxicity of switchable Pres was further validated by MTT assay (Figure 3c; Figure S28, Supporting Information), possibly leading to the ICD of 4T1 cells.

The adenosine triphosphate (ATP) level in 4T1 cells treated with unlocked Pres was determined to be the lowest (Figure 3d; Figure S29, Supporting Information), indicating the release of the highest amount of ATP into environment as “find me” signals. In addition, calreticulin (CRT) exposure in Pres+NIR group was detected to be the highest (Figure 3e,g; Figures S30 and S31, Supporting Information), implying the release of the strongest “eat me” signals. Furthermore, high mobility group box 1 (HMGB1) remained in 4T1 cells was the lowest (Figure 3f,h; Figures S32 and S33, Supporting Information), suggesting that the most of them had been released to stimulate myeloid immune cells.

### 2.6 Switchable Pres Activated DCs via ICD Induction

Since Pres had effectively caused the ICD of 4T1 cells, released signals would then stimulate APCs to activate immune responses. Significantly higher proportions of CD11c^+^CD40^+^ (Figure 3i; Figure S34, Supporting Information), CD11c^+^CD80^+^ (Figure 3j; Figure S35, Supporting Information), CD11c^+^CD83^+^ (Figure 3k; Figure S36, Supporting Information), and CD11c^+^CD86^+^ (Figure 3l; Figure S37, Supporting Information) bone marrow dendritic cells (BMDCs) were simultaneously detected in Pres+NIR group than the others, suggesting that 4T1 cells with unlocked Pres pre‐treatments instigated the highest increase of activated dendritic cells (DCs), indicating the strongest effect of switchable Pres on DCs activation.

### 2.7 Pres was Equipped with Multimodal Imaging Property

**Figure 4:**
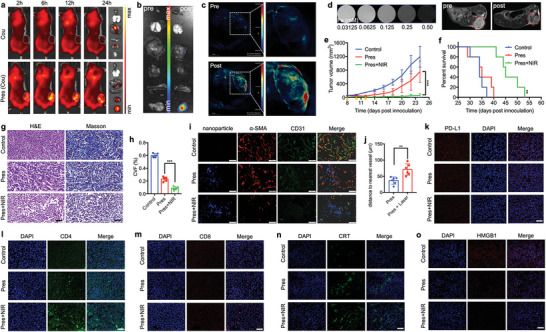
Magnetic targeting and multimodal imaging guided Pres regressed tumor environment through rarefying vessels, loosening structure, improving penetration depth and inversely reprogramming immune microenvironment against carcinoma. a) Biodistribution of Pres. The ex vivo organs (heart, lung, liver, spleen, kidneys and tumor from top to bottom) in the right row were observed at 24 h. b) Fluorescence signal distribution of Pres in ex vivo organs (heart, lung, liver, spleen, kidneys and tumor from top to bottom) pre‐ and 6 h post‐ injection and guidance. c) PAI of Pres pre‐ and 6 h post‐ injection and guidance. d) MRI images of different Pres concentrations in vitro (left) and of tumors (red circles) pre‐ and 6 h post‐ injection and guidance in vivo (right). e) Tumor growth curves of subcutaneous 4T1 tumors (n = 5). The yellow arrows indicated treatments. f) Survival curve (n = 5). g) H&E and Masson staining of ex vivo tumors. Scale bar, 50 µm. h) CVFs determined by Image J. i) Nanoparticle (blue), α‐SMA high expression (red) and CD31 positive (green) cells distributions in ex vivo tumors. Scale bar, 50 µm. j) Distances of nanoplatform signal dots to nearest vessels in i determined by Caseviewer (n = 6). k) PD‐L1 expression in tumors. Scale bar, 100 µm. l,m) CD4^+^ and CD8^+^ cells in deep tumor areas, respectively. Scale bar, 100 µm. n,o) CRT and HMGB1 expression in tumors, respectively. Scale bar, 100 µm. Statistical significance was determined by one‐way ANOVA with Tukey's HSD multiple comparisons post‐hoc test or Student's t‐test. All data are presented as mean ± SD, ** p < 0.01, *** p < 0.001.

Before assessment of in vivo activities, multimodal imaging of Pres was evaluated. To begin with, the biodistribution of Pres, loaded with coumarin (Cou) instead of Su, was conducted at certain time intervals post intravenous injection (*i.v*.) and magnetic targeting. Images in **Figure** **4**a revealed that Pres rapidly accumulated in tumor areas and displayed the strongest signals at 6 h post injection (Figure S38, Supporting Information). Accumulated Pres in tumor area exhibited obviously stronger fluorescence signals at 6 h (Figure 4b). Furthermore, Pres also exhibited excellent photoacoustic imaging (PAI) property showing the margin and main body of solid tumors (Figure 4c), and T_2_ magnetic resonance imaging (MRI) property, with a relaxation rate of 218.37 mM^−1^ s^−1^ (Figure S39, Supporting Information), showing observably darker image of tumor area (Figure 4d) at 6 h. These findings revealed that Pres was endowed with brilliant multimodal imaging property that could facilitate and guide tumor environment regression therapy.

### 2.8 Pres Significantly Inhibited Tumor Growth and Prolonged Survival Time

With the instruction of multimodal imaging, irradiation of Pres was performed at 6 h post injection and magnetic guidance. in vivo treatments in subcutaneous TNBC mice models revealed that unlocked Pres significantly inhibited tumor growth (Figure 4e; Figures S40 and S41, Supporting Information), ending up in the smallest in sizes and the lightest in weights after treatments (Figure S42, Supporting Information). Accordingly, no metastasis was observed in the lungs and livers from mice treated with Pres and irradiation, while many in those treated with the others (Figure S43, Supporting Information). Meanwhile, no obvious adverse effects in heart, kidneys, spleen and mice body weights was noted (Figures S43 and S44, Supporting Information). Furthermore, significantly longer survival time of unlocked Pres treated mice was recorded (Figure 4f; Figure S45, Supporting Information). It was worth noting that no notable difference was observed in the inhibition of tumor growth and metastasis, and elongation of survival time, in the groups treated with Su@PDA or Fe_3_O_4_@PDA with irradiation when compared with those in control group (Figures S41–S43 and S45, Supporting Information), indicating that either inhibiting neovascularization or eliminating CAFs alone, without collaboratively regressing all the main tumor environment characteristics, could not effectively inhibit TNBC progression and aggression. These results evidenced that our switchable Pres achieved excellent antitumor effect in vivo, probably due to the tumor environment regression of TNBC.

### 2.9 Pres Loosened the Stiff Structure of TNBC through Tumor Environment Regression

Hematoxylin‐eosin (H&E) staining of tumor tissues in Figure 4g and Figure S46 (Supporting Information) revealed an observably looser structure of TNBC in Pres+NIR group. Masson staining revealed a significantly looser structure and less collagen deposition in tumors with unlocked Pres treatments (Figure 4g; Figure S47, Supporting Information), in which the collagen volume fraction (CVF) was determined to be the lowest of all (Figure 4h; Figure S48, Supporting Information). Furthermore, a markedly higher proportion of new vessels and CAFs were destroyed by unlocked Pres, leading to the lowest number of CD31 positive cells and α‐SMA high expression cells observed in Figure 4i and Figure S49 (Supporting Information). These findings proved that switchable Pres successfully regressed tumor environment from stiff to loose through antiangiogenesis in cooperation with CAFs and collagen elimination to remove the supports of tumor cells, thus transforming “indestructible fortress” to “vulnerable village”.

### 2.10 Pres Penetrated Deep into TNBC through Tumor Environment Regression

The shift of TNBC from stiff to loose rendered tumor cells with high chance to be exposed to dangers without protection since drugs could now dive freer in tumor tissues. By comparing the number of blue fluorescence signals in tumor tissues (Figure 4i; Figure S49, Supporting Information) and analyzing the distances of these signals to their nearest vessels (Figure 4j; Figure S50, Supporting Information), tumor penetration depth of nanoplatforms could be assessed. Results proved that much more Pres traveled significantly deeper in tumor environment than the others, indicating promoted penetration depth of Pres in TNBC through tumor environment regression.

### 2.11 Pres Reversely Reprogramed Immune Microenvironment via Tumor Environment Regression

Apart from tumor environment regression, switchable Pres was also observed to have relieved immunosuppression from PD‐L1 by CAFs subjugation. PD‐L1 expression in ex vivo tumors of Pres+NIR group was the lowest of all (Figure 4k; Figure S51, Supporting Information). Meanwhile, many more CD4^+^ cells (Figure 4l; Figure S52, Supporting Information) and CD8^+^ cells (Figure 4m; Figure S53, Supporting Information) were observed in the deep tumor environment with unlocked Pres treatments, suggesting that significantly more CD4^+^ cells and CD8^+^ cells had been infiltrated into the deep tumor environment through loosened tumor structure. Furthermore, the highest CRT signals (Figure 4n; Figure S54, Supporting Information) and the lowest HMGB1 signals (Figure 4o; Figure S55, Supporting Information) in deep tumor areas were also detected in Pres+NIR group, indicating that remarkably stronger ICD of tumor cells was induced by Pres in the deep loosened tumor environment. These findings verified that, thanks to tumor environment regression, switchable Pres inversely reprogrammed the tumor immune microenvironment by relieving immunosuppression from PD‐L1, facilitating CD4^+^ cells and CD8^+^ cells infiltration and instigating effective ICD of tumor cells.

### 2.12 Switchable Pres Unleashed Vigorous Systemic Immune Responses in TNBC

**Figure 5:**
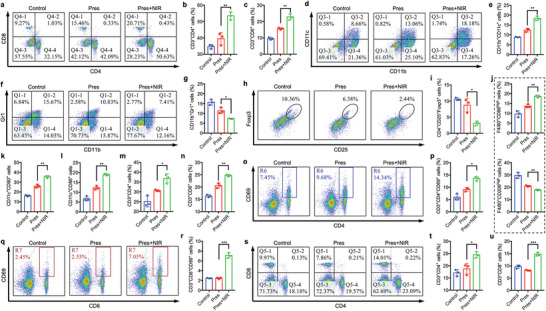
Switchable Pres unleashed vigorous systemic immune responses. a–j) FCM analysis of immune cells in tumor tissues of different groups. a, Infiltration of CD3^+^CD4^+^ cells and CD3^+^CD8^+^ cells. b,c) Quantitative results of CD3^+^CD4^+^ and CD3^+^CD8^+^ T cells in a respectively. d,e) FCM and quantitative analysis of infiltrated DCs. f,g) FCM and quantitative results of MDSCs population. h,i) FCM analysis and quantitative results of Treg population. j) Quantitative analysis of M1 TAM (top) and M2 TAM (bottom) populations. k‐r) FCM analysis of immune cells in lymph nodes of different groups. k,l) Quantitative analysis of DCs activation by CD11c^+^CD80^+^ or CD11c^+^CD86^+^, respectively. m,n) Quantitative results of CD3^+^CD4^+^ and CD3^+^CD8^+^ T cells, respectively. o,p) FCM and quantitative results of CD3^+^CD4^+^CD69^+^ T cells, respectively. q,r) FCM and quantitative results of CD3^+^CD8^+^CD69^+^ T cells, respectively. s–u) FCM analysis of immune cells in spleen of different groups. s, FCM analysis of CD3^+^CD4^+^ and CD3^+^CD8^+^ T cells. t,u) Quantitative results of CD3^+^CD4^+^ and CD3^+^CD8^+^ T cells, respectively. Statistical significance was determined by one‐way ANOVA with Tukey's HSD multiple comparisons post‐hoc test. All data are presented as mean ± SD, *n* = 3, * *p* < 0.05, ** *p* < 0.01, *** *p* < 0.001.

The immune responses in tumor‐bearing mice were subsequently analyzed. Similar with the results in Figure 4l,m, tumors with unlocked Pres treatments were observed with significantly higher proportion of infiltrated CD3^+^CD4^+^ cells (**Figure** **5**a,b) and CD3^+^CD8^+^ cells (Figure 5a,c) by FCM analysis. Accompanied with improved infiltration of helper and cytotoxic T cells, recruitment of DCs was observed to have notably increased as well (Figure 5d,e). Moreover, immunosuppression in TNBC was considerably relieved. Myeloid‐derived suppressor cells (MDSCs) in Pres+NIR group were remarkably less than the others (Figure 5f,g). Meanwhile, unlocked Pres obviously decreased the population of Treg (Figure 5h,i) and M2 tumor associated macrophages (TAMs) (Figure 5j; Figure S56, Supporting Information), but increased that of M1 TAMs (Figure 5j; Figure S57, Supporting Information) in TNBC in vivo. These results further evidenced that switchable Pres had successfully reversed tumor immune microenvironment by tumor environment regression therapy.

Furthermore, an observably higher proportion of DCs had been activated in the lymph nodes of tumor‐bearing mice treated with Pres and irradiation (Figure 5k,l). In addition, significantly higher proportions of helper and cytotoxic T cells were detected (Figure 5m,n; Figure S58, Supporting Information) and had been activated (Figure 5o–r) in Pres+NIR group. Higher increase in helper and cytotoxic T cells proliferation had also been counted in spleens with unlocked Pres treatments (Figure 5s–u). Besides, impressively decline in Th2 cytokines (interleukin (IL)−10, IL‐6, TGF‐β) and increase in Th1 cytokines (IL‐2, interferon (IFN)‐γ, IL‐12a) were noted in serums from tumor‐bearing mice post Pres and irradiation treatments (Figure S59, Supporting Information). These findings confirmed switchable Pres to be an efficient trigger for strong systemic immunotherapy attributed to tumor environment regression in TNBC.

### 2.13 Switchable Pres Inhibited Tumor Growth of Distant Tumors and Generated Efficient Immune Memory in TNBC

**Figure 6:**
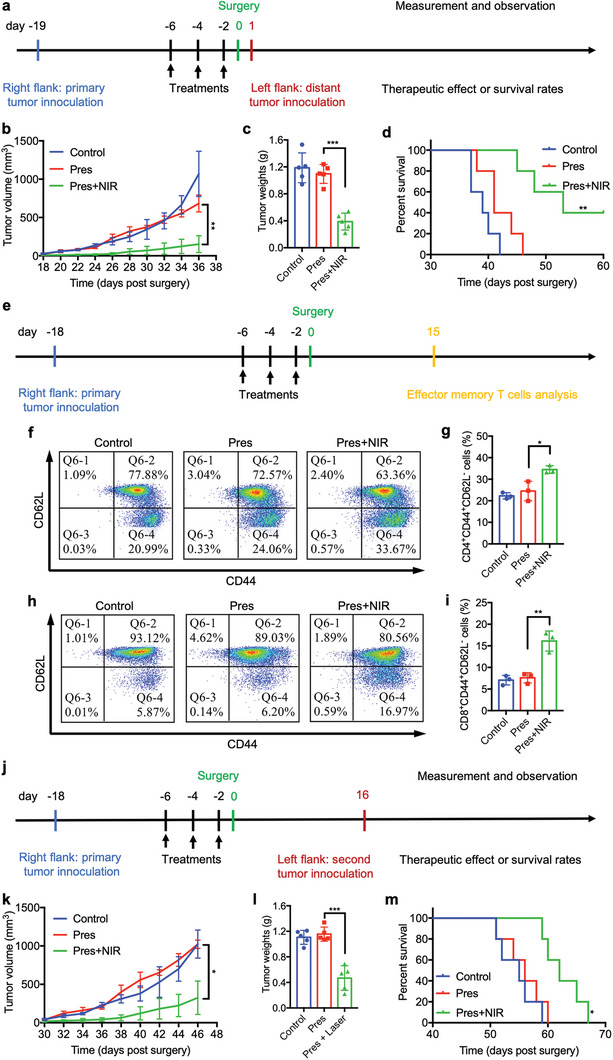
Switchable Pres inhibited tumor growth of distal tumors and generated efficient immune memory via tumor environment regression therapy. a) Illustration of the time axis for experiments of abscopal effect. b) Tumor growth curve of distant tumors (*n* = 5). c) Weights of ex vivo distant tumors (*n* = 5). d) Survival percentage analysis of tumor bearing mice with different treatments in abscopal models (*n* = 5). e) Illustration of the time axis for experiments of T_EM_ cells analysis. f‐i) FCM analysis and corresponding quantitative of CD4^+^ (f,g) and CD8^+^ (h,i) T_EM_ cells denoted as CD3^+^CD4^+^CD44^+^CD62L^−^ cells and CD3^+^CD8^+^CD44^+^CD62L^−^ cells at day 15 after surgery (*n* = 3), respectively. j) Illustration of the time axis for immune memory experiments. k) Tumor growth curve of second tumors (*n* = 5). l) Weights of ex vivo second tumors (*n* = 5). m) Survival percentage analysis of tumor bearing mice with different pre‐treatments in immune memory experiments (*n* = 5). Statistical significance was determined by one‐way ANOVA with Tukey's HSD multiple comparisons post‐hoc test or Student's *t*‐test. All data are presented as mean ± SD, * *p* < 0.05, ** *p* < 0.01, *** *p* < 0.001.

To examine the effect of systemic immune responses triggered by switchable Pres, bilateral subcutaneous TNBC mouse models were established and treated according to the time axis in **Figure** **6**a. The growth of distant tumors in Pres+NIR group was significantly inhibited (Figure 6b,c; Figure S60, Supporting Information), resulting in a remarkably elongated survival time and improved survival rate (Figure 6d), suggesting that systemic immune responses triggered by unlocked Pres displayed strong abscopal effect in TNBC.

Moreover, the effector memory T (T_EM_) cells in spleen post treatments were also analyzed according to the time axis in Figure 6e. Considerably higher levels of CD3^+^CD4^+^CD44^+^CD62L^−^ cells (Figure 6f,g) and CD3^+^CD8^+^CD44^+^CD62L^−^ cells (Figure 6h,i) were detected in Pres+NIR group by FCM analysis on day 15 post treatments and surgery. In addition, observably increased Th1 cytokines (IFN‐γ, IL‐12a and IL‐2) and decreased Th2 cytokines (IL‐6, IL‐10 and TGF‐β) in serums were also detected in tumor‐bearing mice post Pres and irradiation treatments (Figure S61, Supporting Information). These results indicated that strong immune memory had been instigated by systemic immune responses triggered by switchable Pres.

Furthermore, the efficiency of this immune memory was then assessed according to the time axis in Figure 6j. Results showed that such immune memory effect endowed unlocked Pres treated mice with efficient capability to inhibit the growth of second tumors (Figure 6k,l; Figure S62, Supporting Information) and to survive longer (Figure 6m). These findings added additional sound evidence for the fact that switchable Pres could induce efficient immune memory against carcinoma through tumor environment regression therapy.

## 3 Conclusion

Here, we defined and implemented tumor environment regression therapy of TNBC by switchable Pres as the first attempt in the current study. Pres directed toward new vessels and CAFs in tumor environment developed from oncogenesis coevolution of tumor cells and surrounding stroma. By inhibiting neovascularization, tumor vessels were rarefied, and additional time was bought for Pres to win the war against carcinoma. By taking advantage of the fact that CAFs, as the barriers and defenses between blood vessels and tumor cells, were inclined to intercept and endocytose intruders,^[14^ ^]^ Pres eliminated CAFs in tumor environment. Eradication of CAFs subsequently reduced collagen deposition. The absence of CAFs and collagens after tumor environment regression made room for the penetration of Pres and lymphocytes. Furthermore, self‐tolerance hijacked by TNBC was then relieved: tumor immune microenvironment, including PD‐L1 expression, immune cells and cytokines, was retrograded to the status as it should be before the coevolution.^[^ ^24^, ^26^, ^46^, ^47^ ^]^ Consequently, immunosuppression was removed, and immune cells were recruited through ICD of tumor cells and rushed deep into the retrograded tumor environment to burst systemic immunoresponses to sweep tumor cells away and generate immune memory to curb tumor progression.

## 4 Experimental Section

### Materials, Cells and Animals

Dopamine hydrochloride, sunitinib and Fe_3_O_4_ nanoparticles were all purchased from Aladdin, Shanghai, China. 4T1 cells (mouse triple‐negative breast cancer cells) were obtained from American Type Culture Collection (ATCC), Manassas, VA, USA. BALB/c mice (female, 6–8 weeks) were purchased from HFK Bio‐Technology Company, Beijing, China. All animal procedures were performed following the protocol of State Key Laboratory of Biotherapy approved (20 230 302 005) by the Institutional Animal Care and Treatment Committee of Sichuan University, Chengdu, China.

### Preparation and Characterization

Briefly, Fe_3_O_4_ nanoparticles (Aladin, Shanghai, China) were dispersed in chloroform with oleic acid under ultrasound. Then, dopamine hydrochloride (Aladin, Shanghai, China) was dissolved in Tris buffer (pH 8.5), and at the same time Su (Aladin, Shanghai, China) was dissolved in chloroform. These solutions were mixed and followed by generous stirring with sonication for at least 6 h. Finally, Pres was obtained by centrifugation to remove the dissociative reagents, and then evaluated by DLS, TEM, SEM, UV–vis‐NIR spectrum, fluorescence scanning, stability assessment, drug release behavior. The contents of PDA, Fe_3_O_4_ and Su in Pres were determined through BCA protein assay, inductively coupled plasma‐atomic emission spectrometry and high‐performance liquid chromatography, respectively.

### Photothermal Properties

Pres and other nanoparticles were suspended in 1 mL water, irradiated and recorded with 808 nm laser (1.0 W cm^−2^) for different times at room temperature. Meanwhile, corresponding experiments of various concentrations of Pres were employed. The in vivo temperature changes and corresponding images of photothermal nanomaterials were recorded. Photothermal conversion stability was also evaluated by on‐off experiment with interval of 5 min for 5 cycles. The photothermal conversion efficiency (η) of Pres was calculated by the previously reported method.^[^ ^49^, ^50^, ^51^ ^]^ Briefly, 1 mL of Pres solution in vials was irradiated under 808 nm laser (1.0 W cm^−2^) till the temperature reached a stable plateau. The temperature of the solutions was monitored in real‐time. And then the laser irradiation was removed. The time constant (τ_s_) of heat transfer was determined by applying the linear time‐dependent data collected during the cooling period. The photothermal conversion efficiency was calculated by following equations:

(1) $$\eta \;=\frac{mc}{\tau_{s}}\Delta T_{max}-Q_{s}/I\left(1-10^{-A_{\lambda }}\right)$$

where m is the mass of water used to suspend the nanoparticles, c is the heat capacity of water, τ_s_ is time constant for heat transfer, Δ*T_max_* is the maximum temperature change, *Q_s_* is relevant to the light absorbance of pure water, *I* is the laser power density, and *A*_λ_ is the absorbance of the solution at 808 nm in the UV‐vis‐NIR spectrum. For Pres, η was calculated to be 38.3%. Similar to the in vitro experiments, the in vivo photothermal property was assessed through intratumorally injecting Pres and other nanoparticles at equivalent dosages, and irradiating with 808 nm laser (1.0 W cm^−2^) for different times at room temperature. The temperature changes in tumor areas were recorded.

### Cellular Uptake In Vitro

Based on the fluorescence character of Pres, cellular uptake experiments in CAF‐like 3T3 cells or 4T1 cells were carried out. Briefly, cells were incubated with PDA, Su@PDA, Fe_3_O_4_@PDA or Pres for 4 h, and then carefully washed and collected for FCM or fluorescence microscopy observation.

### Cytotoxicity In Vitro

The antitumor effect of Pres was evaluated by MTT assay and live‐dead staining. Briefly, 4T1 cells or CAF‐like 3T3 cells were incubated with medium (control), Su, Fe_3_O_4_, PDA, Su@PDA, Fe_3_O_4_@PDA, or Pres at various corresponding concentrations for 4 h, then washed and exposed or not exposed to laser (1.0 W cm^−2^, 5 min). After 24 h of incubation, cell viability was measured following the standard method of MTT or live‐dead staining (KeyGEN, Nanjing, China).

### Antiangiogenesis In Vitro

Antiangiogenesis effect was evaluated through wound healing, tube formation and invasion assays. For wound healing experiment, HUVEC cells were seeded into 12‐well plates. After scratches were made, cells were treated with different formulas with or without irradiation as mentioned. After 12 h, the scratches were observed under microscope. For tube formation, HUVEC cells were seeded into 96‐well plates pre‐paved with Matrigel, and then followed with different treatments as mentioned. After 8 h, the cells were observed under microscope. For invasion assay, HUVEC cells were seeded into 12‐well trans‐wells pre‐paved with Matrigel in DMEM medium without FBS, and put into 12‐well plates with DMEM medium with 10% FBS. The cells were then followed with different treatments as mentioned. After 16 h, the trans‐wells were carefully washed to remove the Matrigel. Invaded cells on the screens were stained with crystal violet and observed under microscope.

### Cancer Associated Fibroblasts Modulation In Vitro

NIH3T3 cells were activated by TGF‐β (final concentration of 10 ng mL^−1^) for 12 h to transform into CAF‐like phenotype. The cellular uptake and intracellular fluorescence of PDA coated nanoparticles were detected by incubating CAF‐like 3T3 cells with different formulas for 4 h and then assessing through microscope or FCM after washing with PBS. Next, MTT assay was performed according to the method as mentioned. Then, the influence of CAF‐like 3T3 cells on the PD‐L1 expression of 4T1 cells was evaluated. Briefly, CAF‐like 3T3 cells were seeded in 6‐well trans‐wells and put into 6‐well plates pre‐paved with 4T1 cells. After incubation for 24 h, PD‐L1 expression of 4T1 was assessed by FCM and Western Blot. Finally, the modulatory effect of Pres to CAF‐like 3T3 cells on the PD‐L1 expression of 4T1 cells were further evaluated through pre‐treating CAF‐like 3T3 cells with different treatments as aforementioned and then putting the 6‐well trans‐wells into 6‐well plates pre‐paved with 4T1 cells. After incubation for 24 h, PD‐L1 expression of 4T1 was assessed by FCM and Western Blot.

### Penetration in Multicellular Hybrid Tumor Spheroids In Vitro

To mimic the solid tumor microenvironment in which tumor cells and fibroblasts grow together, 4T1 and CAF‐like 3T3 cells cocultured hybrid tumor spheroids were established. Briefly, 60 µL of hot 2.0% (w/v) agarose solution was added to 96‐well plates and then cooled to room temperature. 4T1 cells were mixed with CAF‐like 3T3 cells at a ratio of 2:1, seeded in 96‐well plates, and cultured for 2–3 days to grow into spheroids. Then, the spheroids were treated with different formulas with or without irradiation as aforementioned. After incubation for 24 h, the integrity of spheroids was observed under microscope to assess the toxicity of formulas on hybrid spheroids. The penetration depth was evaluated through incubating the spheroids with PDA coated nanoparticles for 6 h, washing with precooled PBS, fixing in 4.0% paraformaldehyde, and then observing with a confocal laser microscope using Z‐axis scanning from the top of spheroids to the middle.

### Immunogenic Cell Death In Vitro

Immunogenic cell death was investigated according to the released ATP and overexpression of CRT and HMGB1. For ATP levels, 4T1 cells seeded in 24‐well plates were incubated with different formulas for 4 h, then washed and irradiated or not. After another 2 h, the cells were collected for ATP detection by ATP assay kit (Beyotime, Shanghai, China). For CRT levels, 4T1 cells were treated as aforementioned, then washed and stained with CRT antibody and Rho‐labeled second antibody. For HMGB1 levels, the staining of HMGB1 antibody and secondary antibody was performed after 4.0% paraformaldehyde and 0.1% Triton X‐100 treatment. The cells were then detected through FCM or CLMS.

### Activation of Dendritic Cells In Vitro

BMDCs were isolated from the thigh bones and tibias of 5–6 weeks old C57BL/6 mice and stimulated with 20 ng mL^−1^ GM‐SCF. The isolated BMDCs were incubated with different formulas for 24 h before FCM analysis. The expressions of CD40, CD80, CD83 and CD86 on treated BMDCs were detected.

### Biodistribution In Vivo

The magnetic targeting ability of Fe_3_O_4_ was assessed through in vivo imaging of small animals. In brief, Cou was loaded into nanoplatforms instead of Su for better fluorescence in vivo. Tumor‐bearing BALB/c mice (*n* = 3) were *i.v*. injected with free Cou and Pres (Cou) at equivalent dosage with a magnet tied to the tumor areas. The fluorescence was detected by Caliper IVIS Lumina II system at different time intervals. At 24 h, the mice were sacrificed to detect the fluorescence in ex vivo hearts, lungs, livers, spleens, kidneys and tumors. The radiant efficiency in tumor areas at different time intervals was statistically compared afterwards.

### Photoacoustic Imaging

In brief, tumor‐bearing BALB/c mice (*n* = 3) were *i.v*. injected with PBS (control) and Pres with a magnet tied to the tumor areas. The PAI was performed at 0 and 6 h after injections. The mean pixel intensity in tumor areas of each group at different time intervals was statistically compared afterwards.

### Magnetic Resonance Imaging

Pres of different Fe concentrations were detected by MRI to assess the T_2_ relaxation time and MRI efficiency in vitro. For in vivo MRI, Tumor‐bearing BALB/c mice (*n* = 3) were *i.v*. injected with PBS (control) and Pres with a magnet tied to the tumor areas before the MRI was performed at 0 and 6 h after injections.

### Antitumor Effect In Vivo

Triple negative breast cancer cell line 4T1 cells were inoculated in the right flanks of BALB/c mice to establish 4T1 subcutaneous models. When the tumor volume reached ≈100 mm^3^, tumor‐bearing mice were randomly divided into 9 groups (*n* = 5), and *i.v*. injected with PBS (control), PDA, Su@PDA, Fe_3_O_4_@PDA, Pres with a magnet tied to the tumor areas on day 8, day 10 and day 12 with or without irradiation. Tumor size (calculated as length × width^−2^) and body weight were monitored every other day. On day 24, all mice were sacrificed, and H&E staining was performed to analyze tissue damage after various treatments.

Based on the administration schedule of in vivo antitumor effect, long‐term survival was recorded in a separate cohort of mice. Mice were euthanized when they reached one of the endpoints: loss of 20% body weight, tumor size > 2000 mm^3^, hunched back, or lethargic.

### Tumor Environment Regression Therapy Assessment

In a separate cohort experiment, tumor‐bearing mice were sacrificed and ex vivo tumors were collected on day 14. Except from H&E staining, the ex vivo tumors were evaluated by Masson staining to analyze collagen deposition after various treatments. Immunofluorescence (IF) staining of α‐SMA and CD31 were performed to assess the anti‐CAFs and antiangiogenesis effects of various treatments. Tumor penetration depth in vivo was analyzed by the distance of PDA fluorescence dot to the nearest vessel. IF staining of PD‐L1 was carried out to evaluate the modulation of CAFs after treatments in vivo, so were CD4 and CD8 to assess the infiltration of T cells in tumor tissues. Moreover, in vivo ICD was investigated by IF staining of CRT and HMGB1 after various treatments.

In a separate experiment to investigate immune responses after treatments, representative mice were randomly divided into 3 groups (*n* = 3), and *i.v*. injected with PBS (control), Pres with a magnet tied to the tumor areas on day 8, day 10 and day 12 with or without irradiation. The mice were sacrificed on day 13. Their blood samples were collected for cytokine detection with LEGENDplex mouse B cell panel standard cocktail according to protocol. Immune microenvironment in tumors, DCs and T cells in lymphonodi, and T cells in spleens were detected by FCM.

### Abscopal Effect In Vivo

4T1 tumor‐bearing mice were established and received treatments as aforementioned. One day after the last injection and irradiation, the primary tumor in the right flank was eliminated by surgery. In the next day, denoted as day 1, 4T1 cells were inoculated in the left flank to establish distant tumors. Tumor size and body weight were monitored every other day. On day 36, all mice were sacrificed and ex vivo tumors were photographed and weighed. Long‐term survival was recorded in a separate cohort of mice.

### Immune Memory In Vivo

Representative mice were treated as aforementioned, and the day to eliminate right flank tumors by surgery was denoted as day 0. On day 15, mice were sacrificed. The blood samples were collected for cytokine detection with LEGENDplex mouse B cell panel standard cocktail according to protocol. T effector memory cells in spleens were detected by FCM. In a separate experiment on day 16, 4T1 cells were inoculated in the left flank. Tumor size and body weight were monitored every other day. Long‐term survival was recorded in another separate cohort of mice.

### Statistical Analysis

Data were presented as means ± standard deviations (SD). Statistical significance was determined by either a one‐way analysis of variance (ANOVA), one‐way ANOVA with Tukey's HSD multiple comparisons post‐hoc test, or two‐tailed Student's t test. Significant differences between groups were indicated by * *p* < 0.05, ** *p* < 0.01 and *** *p* < 0.001. Sample sizes were carefully determined based on preliminary data or previous experiments. The number of independent repetitions for each experiment was presented in the main text or in the figure legends.

Statistical calculations were performed mainly by GraphPad Prism version 7. WB statistical analysis of PD‐L1 expression was compared by the relative integrated density calculated via ratios of the gray value of PD‐L1 to that of GAPDH in each group by Image J. Immunofluorescence statistical analysis was carried out by two independent investigators randomly chose 6 equal‐sized fields for assessment in a blinded fashion. Nanoplatform penetration depth was determined according to the distance of nanoplatform signal dot (n = 6) to the nearest vessels by Caseviewer.

## Conflict of Interest

The authors declare no conflict of interest.

## Author Contributions

X.Z.H., Q.J.W. and C.Y.G. conceived the concept. X.Z.H. designed and performed the experiments. L.L., C.Q.O. and L.G. helped in animal model establishments, treatments and results recordings. M.L.S. helped with immunohistochemical staining. M.L.S., X.C.L., M.M.Z., R.W. and X.R.K. helped in tumor immune microenvironment detection. F.R.L. and R.L. helped with multimodal imaging. X.Z.H. and L.L. performed data interpretation and analysis. X.Z.H. wrote the manuscript and all the authors discussed the results and contributed to writing portions of the manuscript and editing the manuscript. Q.J.W. and C.Y.G. supervised these works. C.Y.G. acquired the funding for these works.

## Supporting information

Supporting Information

## Data Availability

The data that support the findings of this study are available from the corresponding author upon reasonable request.
