# Supplementary material for: Tumor Environment Regression Therapy Implemented by Switchable Prune‐to‐Essence Nanoplatform Unleashed Systemic Immune Responses
Source: Adv Sci (Weinh). 2023 Oct 24;10(35):2303715. doi: 10.1002/advs.202303715 (PMC10724435; doi:10.1002/advs.202303715)
Supplement: Supplementary file 1 — Supporting Information [file ADVS-10-2303715-s001.pdf]

ADVANCED SCIENCE
Open Access

Supporting Information

for *Adv. Sci.*, DOI 10.1002/advs.202303715

Tumor Environment Regression Therapy Implemented by Switchable Prune-to-Essence Nanoplatform Unleashed Systemic Immune Responses

*Xianzhou Huang, Lu Li, Chunqing Ou, Meiling Shen, Xinchao Li, Miaomiao Zhang, Rui Wu, Xiaorong Kou, Ling Gao, Furong Liu, Rui Luo, Qinjie Wu and Changyang Gong**

Supporting Information for

Tumor Environment Regression Therapy Implemented by Switchable Prune-to-essence Nanoplatform Unleashed Systemic Immune responses

Xianzhou Huang[1], Lu Li[1], Chunqing Ou[1], Meiling Shen[1], Xinchao Li[1], Miaomiao Zhang[1], Rui Wu[1], Xiaorong Kou[1], Ling Gao[2], Furong Liu[1], Rui Luo[1], Qinjie Wu[1], Changyang Gong[1,]*

[1] Department of Biotherapy, Cancer center and State Key Laboratory of Biotherapy, West China Hospital, Sichuan University, Chengdu 610041, China

[2] Department of Medical Oncology, Cancer Center, West China Hospital, Sichuan University, Chengdu 610041, China

* Corresponding authors (C Gong). Correspondence to: Department of Biotherapy, Cancer Center and State Key Laboratory of Biotherapy, West China Hospital, Sichuan University, Chengdu 610041, China

E-mail addresses: chygong14@163.com.

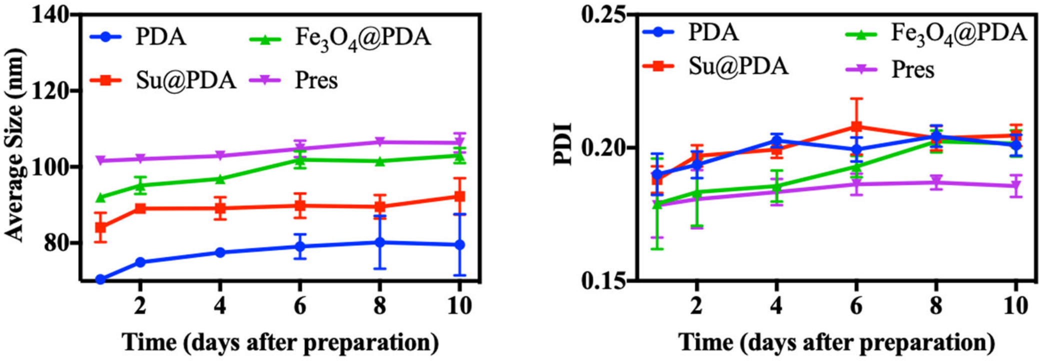

Supplementary Fig. 1. Average size and PDI of nanoplatforms in PBS monitored for 10 days (n=3).

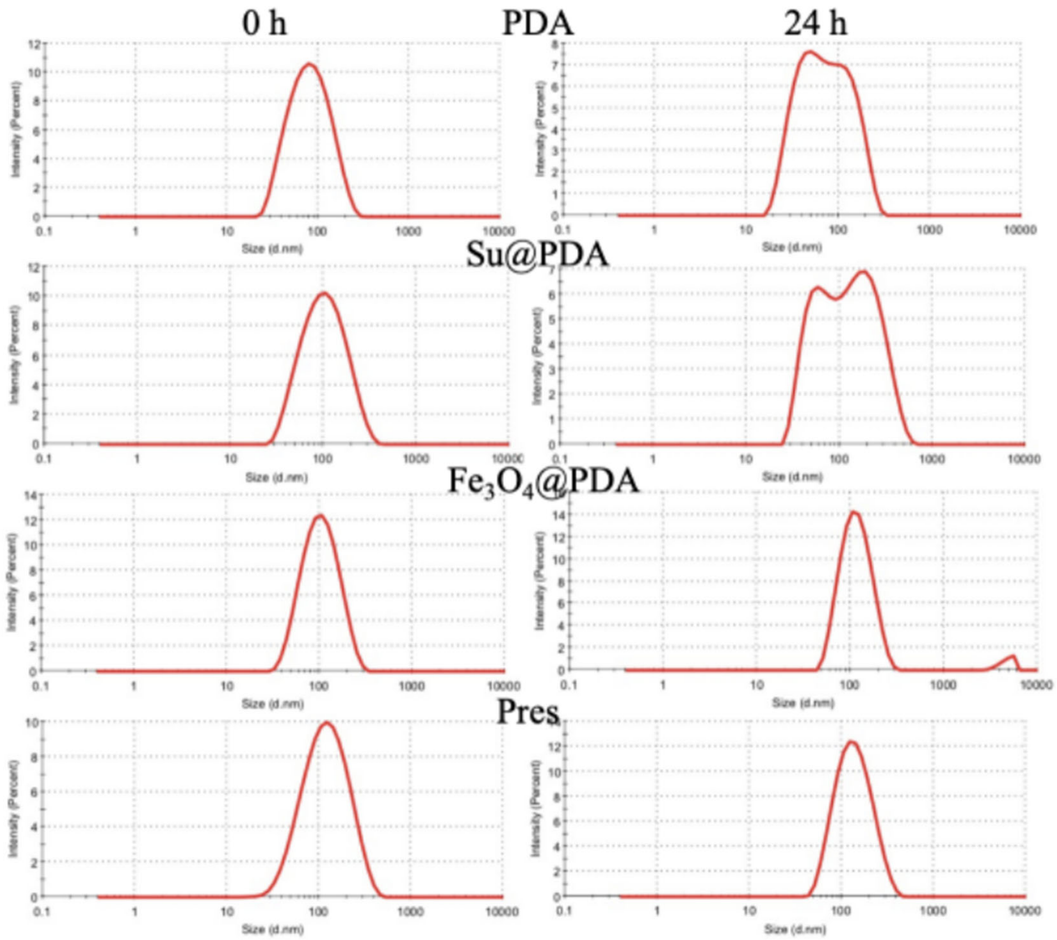

Supplementary Fig. 2. Size distribution of Pres, PDA, Su@PDA and Fe_3O_4@PDA before and after dispersion in 10% FBS for 24 h, respectively.

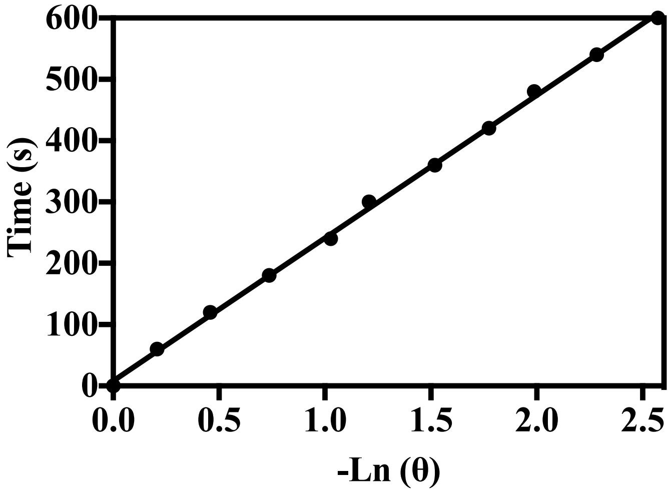

Supplementary Fig. 3. τ_s was 232.84 s, calculated from the slope of Cooling time vs -Ln(θ).

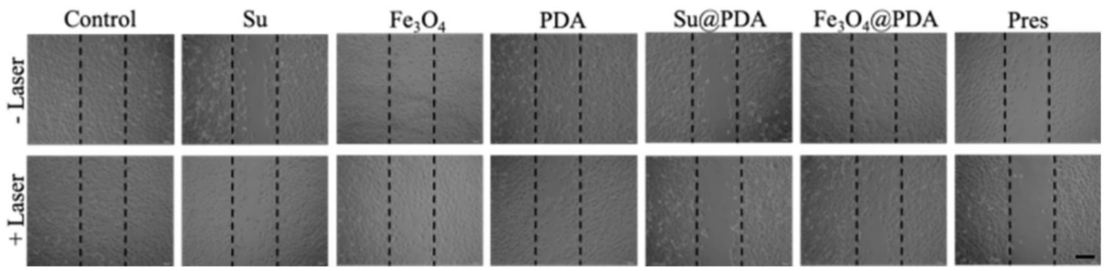

Supplementary Fig. 4. Images of wound healing of HUVEC cells. Dotted lines represented the original wound margins. Scale bar, 400 μm.

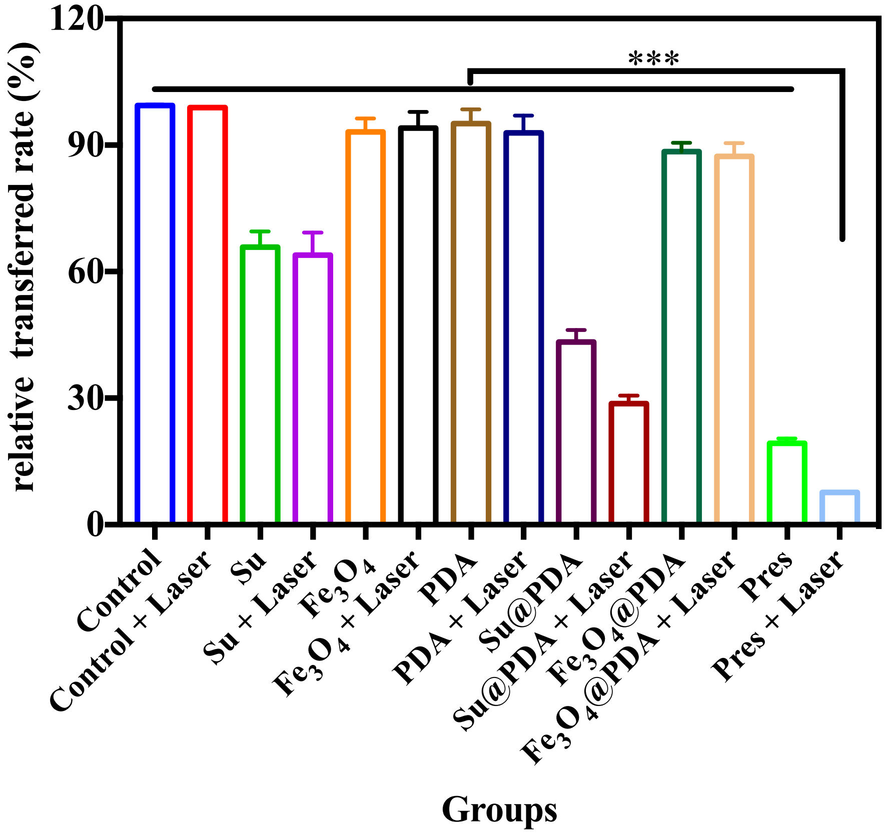

Supplementary Fig. 5. Quantitative analysis of wound healing of HUVEC cells, Su-containing groups: Su, Su+laser, Su@PDA, Su@PDA+laser, Pres, Pres+laser, non-Su groups: control, control+laser, Fe_3O_4, Fe_3O_4+laser, PDA, PDA+laser, Fe_3O_4@PDA, Fe_3O_4@PDA+laser, n=3, *** $p < 0.001$.

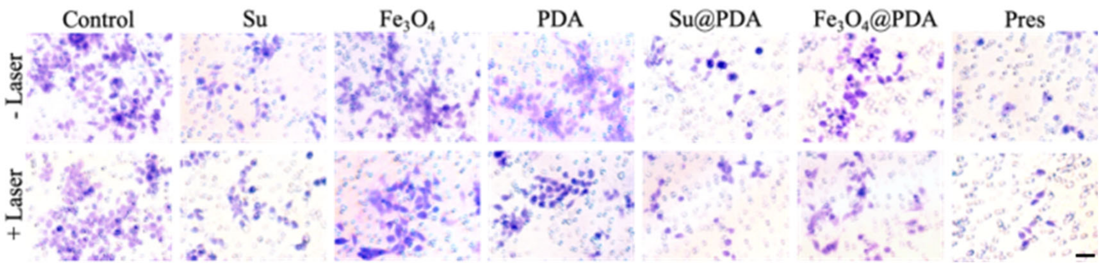

Supplementary Fig. 6. Images of invasion assay of HUVEC cells. Scale bar, 200 μm.

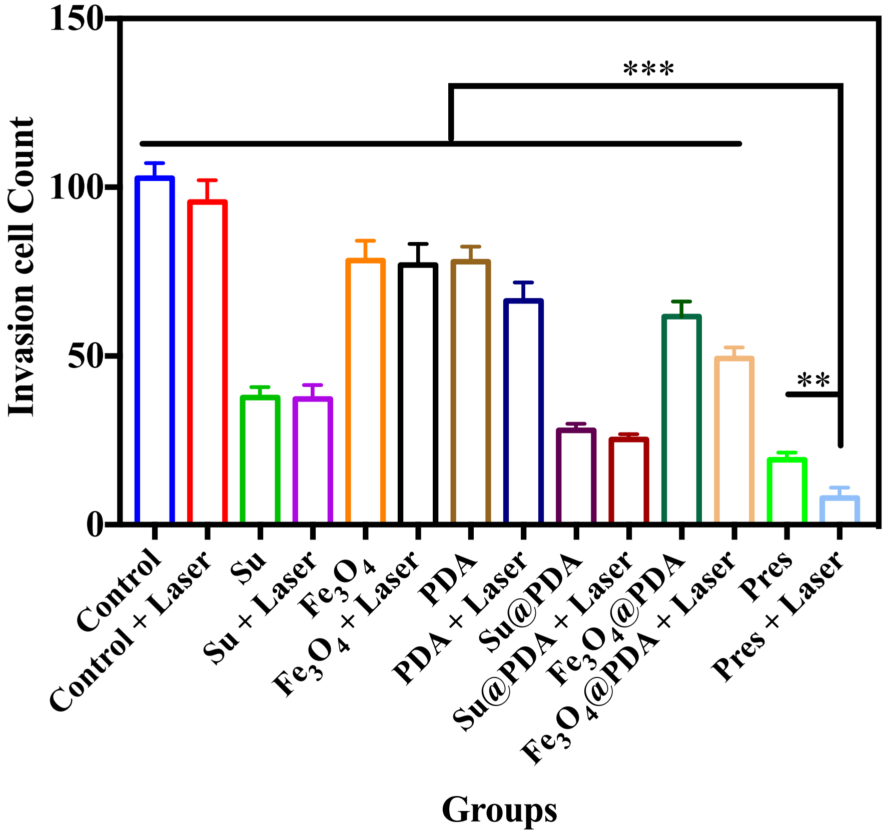

Supplementary Fig. 7. Quantitative analysis of invasion assay of HUVEC cells. n=3, ** $p < 0.01$, *** $p < 0.001$.

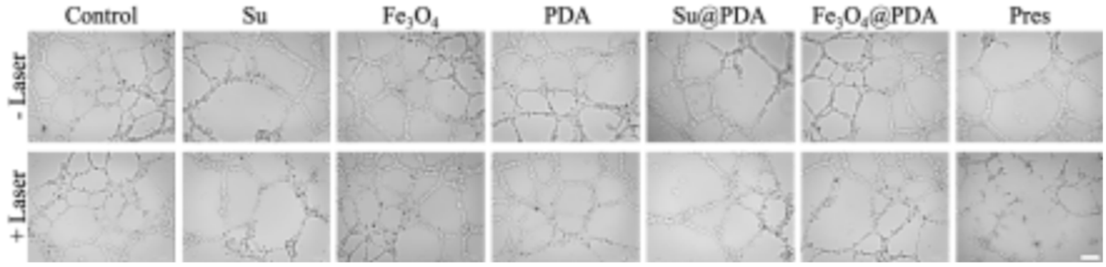

Supplementary Fig. 8. Images of tube formation of HUVEC cells. Scale bar, 400 μm.

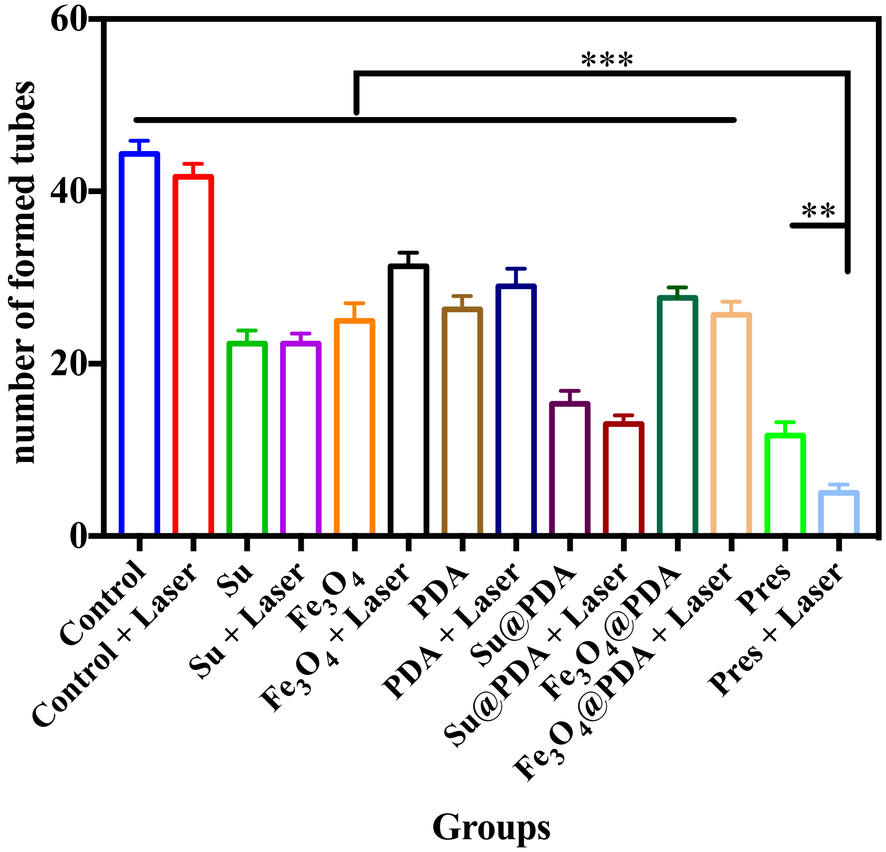

Supplementary Fig. 9. Quantitative analysis of tube formation of HUVEC cells. n=3, ** $p < 0.01$, *** $p < 0.001$.

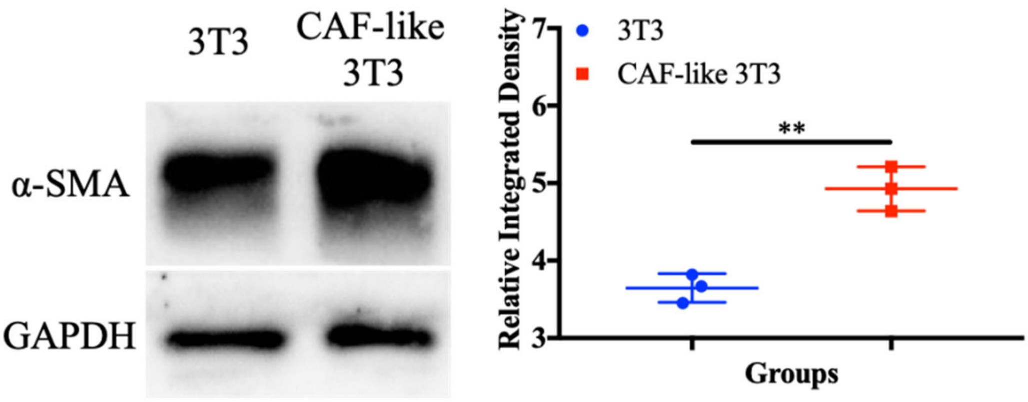

Supplementary Fig. 10. WB results and quantitative analysis of α-SMA expression in 3T3 cells before and after induction with TGF-β overnight. n=3, ** $p < 0.01$.

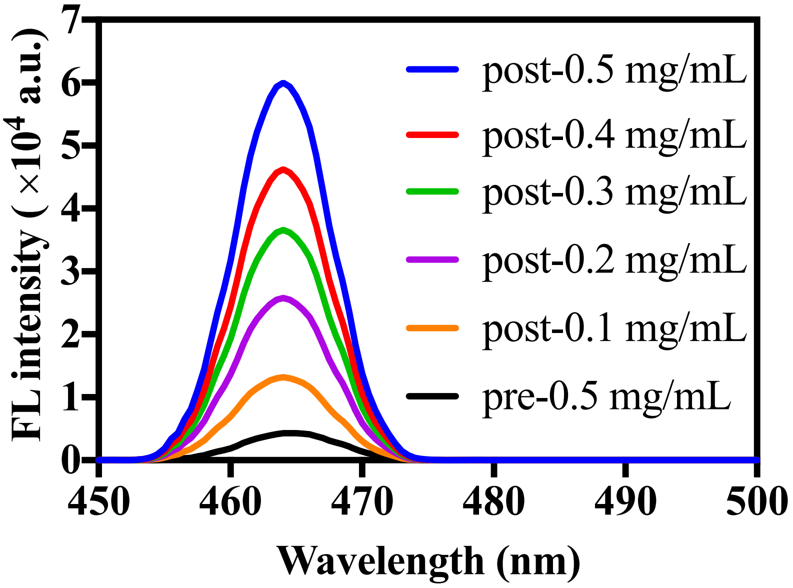

Supplementary Fig.11. Fluorescence intensity of Pres pre- and post- degradation by 30% H_2O_2 for 5 min.

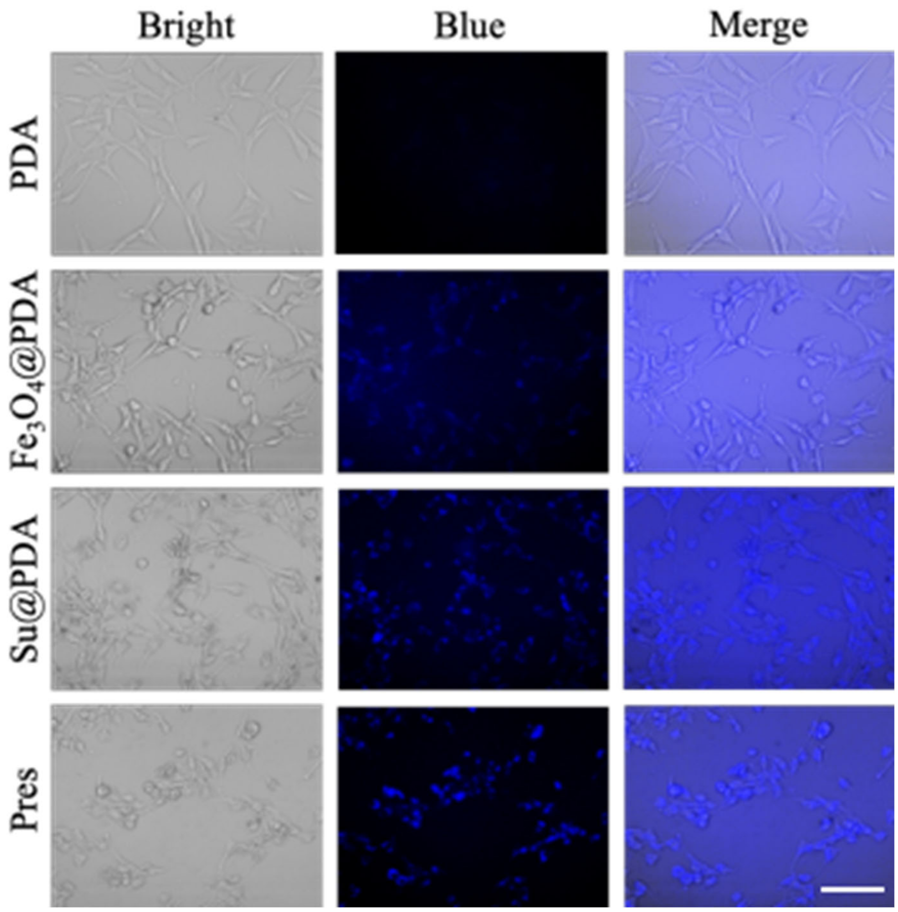

Supplementary Fig. 12. Fluorescence microscope images of cellular uptake analysis on CAF-like 3T3 cells. Blue fluorescence indicated the presence of PDA component. Scale bar, 100 μm.

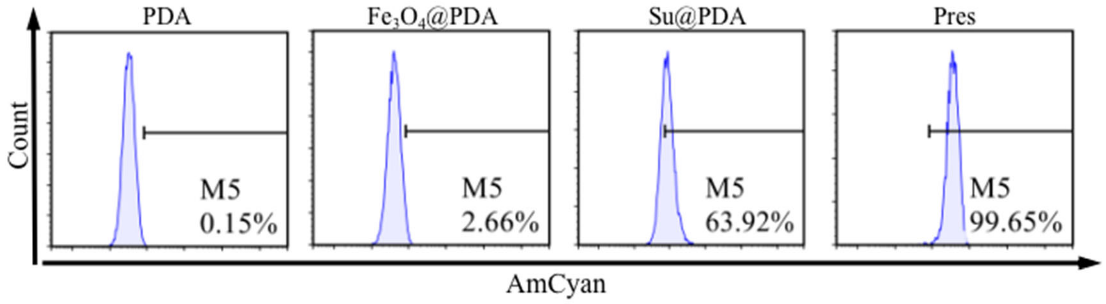

Supplementary Fig. 13. Cellular uptake in CAF-like 3T3 cells detected by FCM.

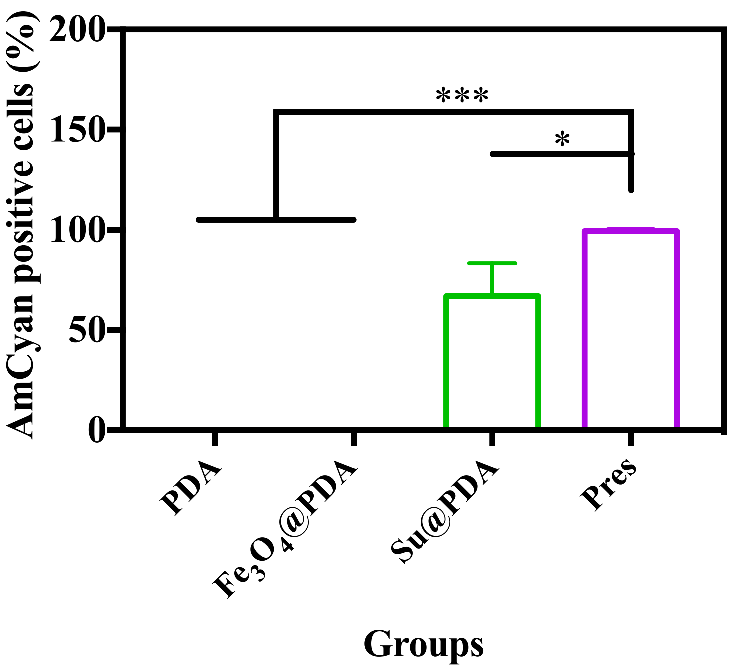

Supplementary Fig. 14. Quantitative analysis of cellular uptake. n=3, * $p < 0.05$, *** $p < 0.001$.

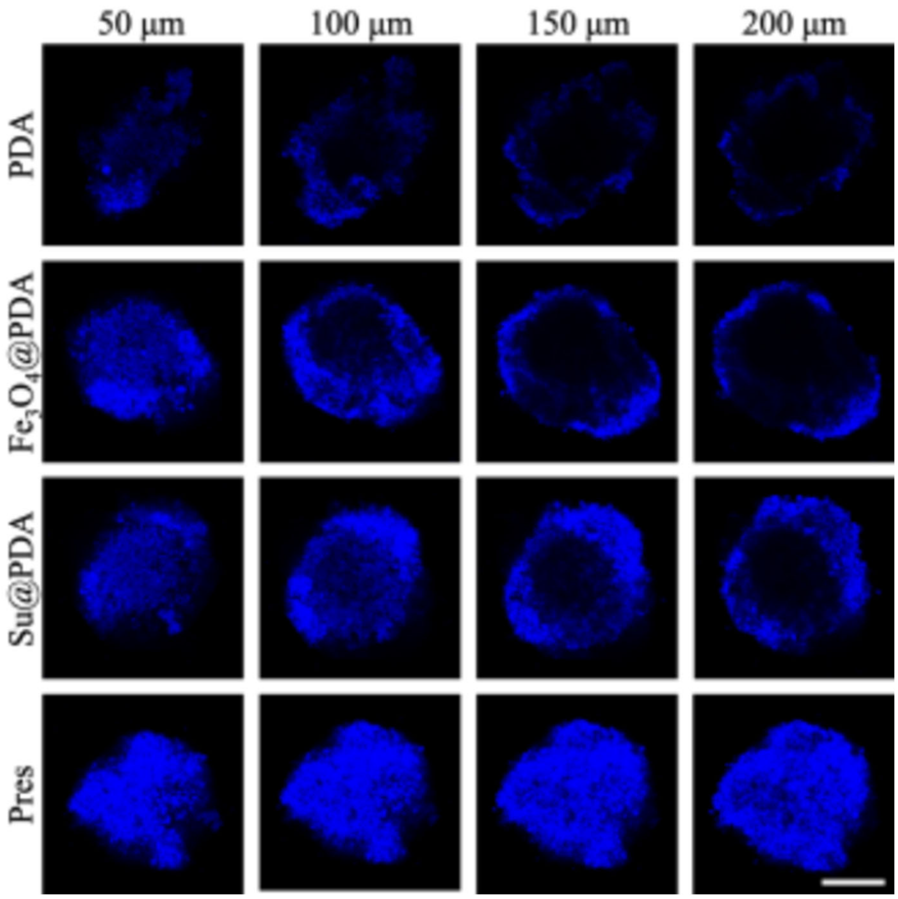

Supplementary Fig. 15. CLSM images of 4T1 and CAF-like 3T3 hybrid spheroids at 50, 100, 150, 200 μm depth sections. Blue signals represented the PDA components in each nanoplatforms.

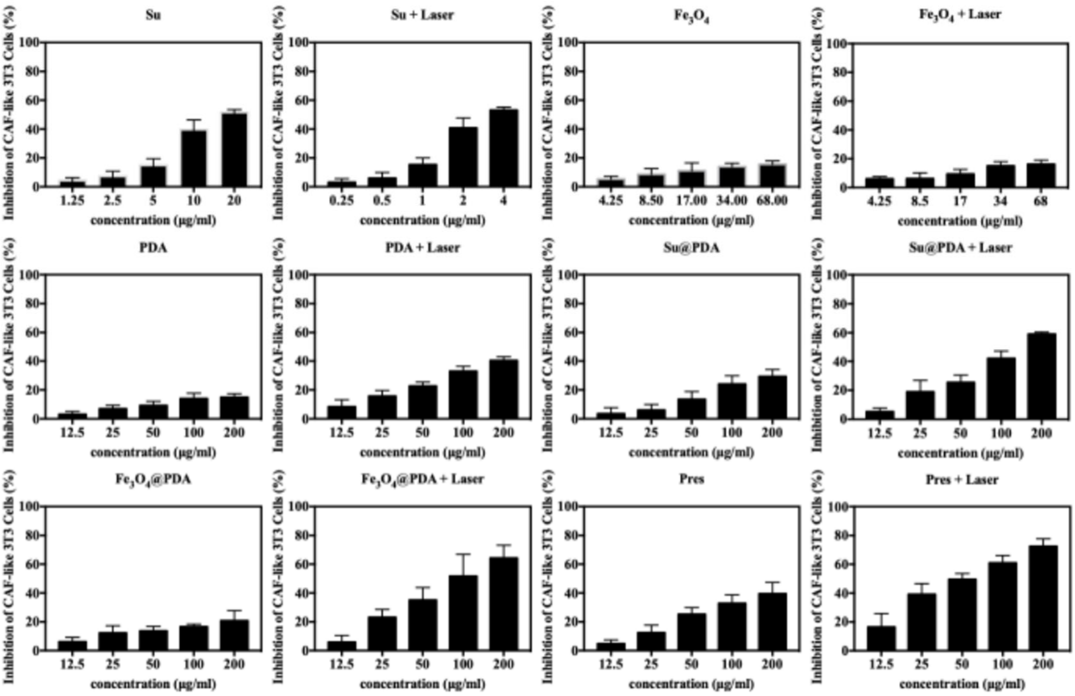

Supplementary Fig. 16. MTT assay on CAF-like 3T3 cells.

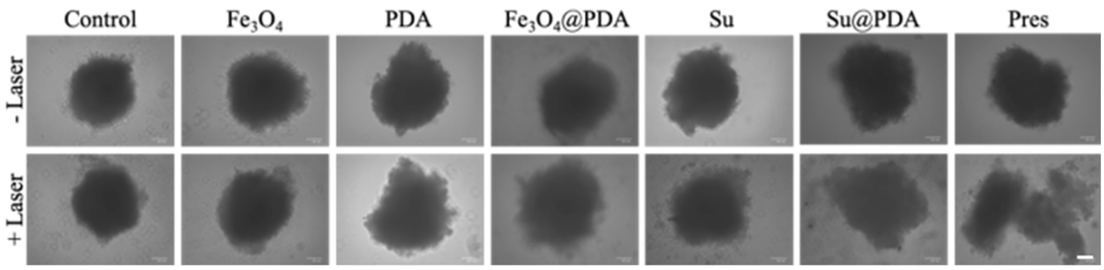

Supplementary Fig. 17. Images of 4T1 and CAF-like 3T3 hybrid spheroids after different treatments. The disassembly of the hybrid spheroid treated with Pres and irradiation indicated loosened structure of hybrid spheroid. Scale bar, 100 μm.

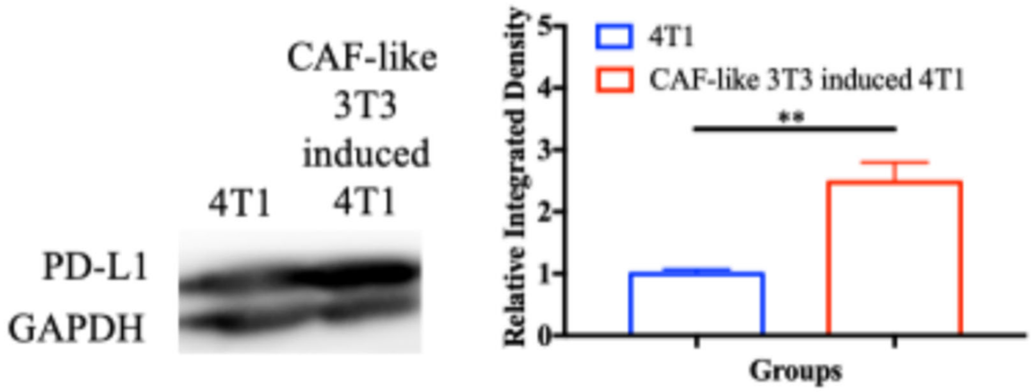

Supplementary Fig. 18. WB results and quantitative analysis of PD-L1 expression in 4T1 cells before and after CAF-like 3T3 cells induction. n=3, ** $p < 0.01$.

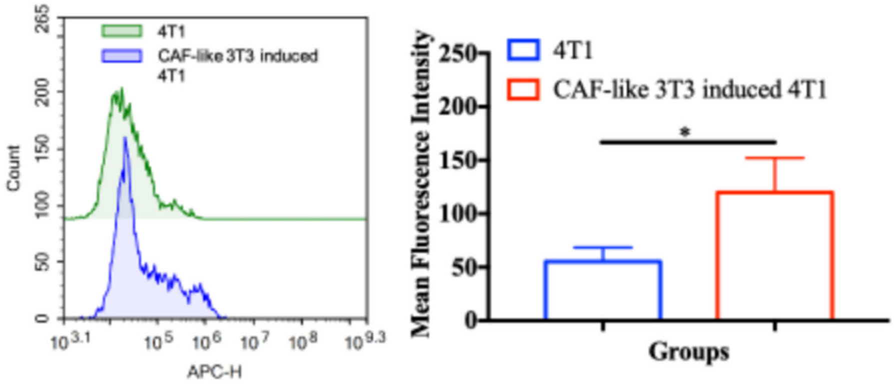

Supplementary Fig. 19. FCM and corresponding quantitative analysis of PD-L1 expression in 4T1 cells before and after CAF-like 3T3 cells induction. n=3, * $p < 0.05$.

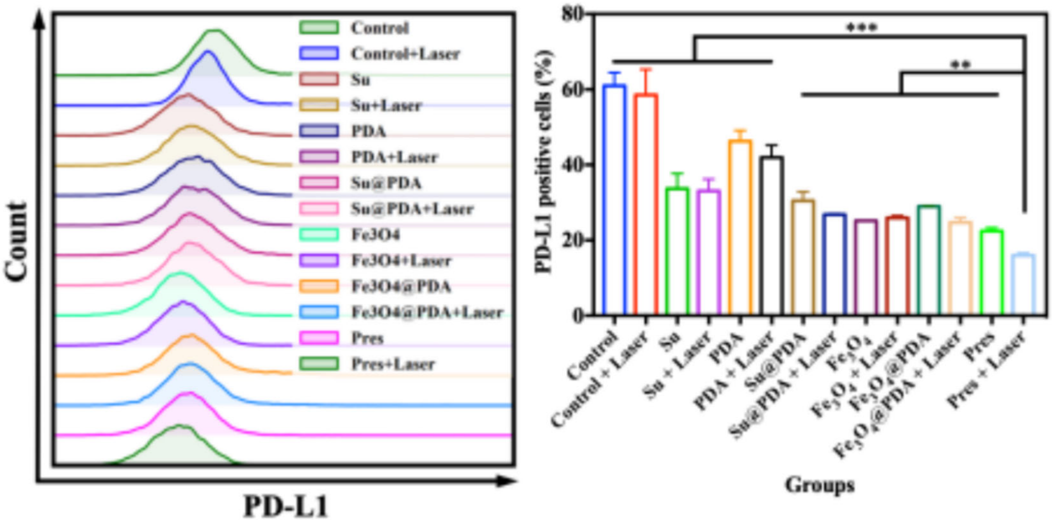

Supplementary Fig. 20. FCM analysis of PD-L1 expression in 4T1 cells induced by CAF-like 3T3 cells with different pretreatments. n=3, ** $p < 0.01$, *** $p < 0.001$.

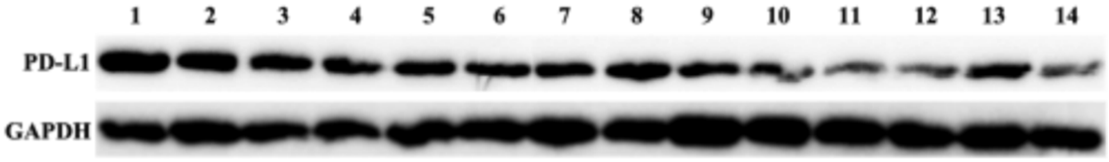

Supplementary Fig. 21. WB bands of PD-L1 and GAPDH expressions in 4T1 cells with different pre-treatments. 1: control, 2: control+laser, 3: Su, 4: Su+laser, 5: Fe_3O_4, 6: Fe_3O_4+laser, 7: PDA, 8: PDA+laser, 9: Su@PDA, 10: Su@PDA+laser, 11: Fe_3O_4@PDA, 12: Fe_3O_4@PDA+laser, 13: Pres, 14: Pres+laser.

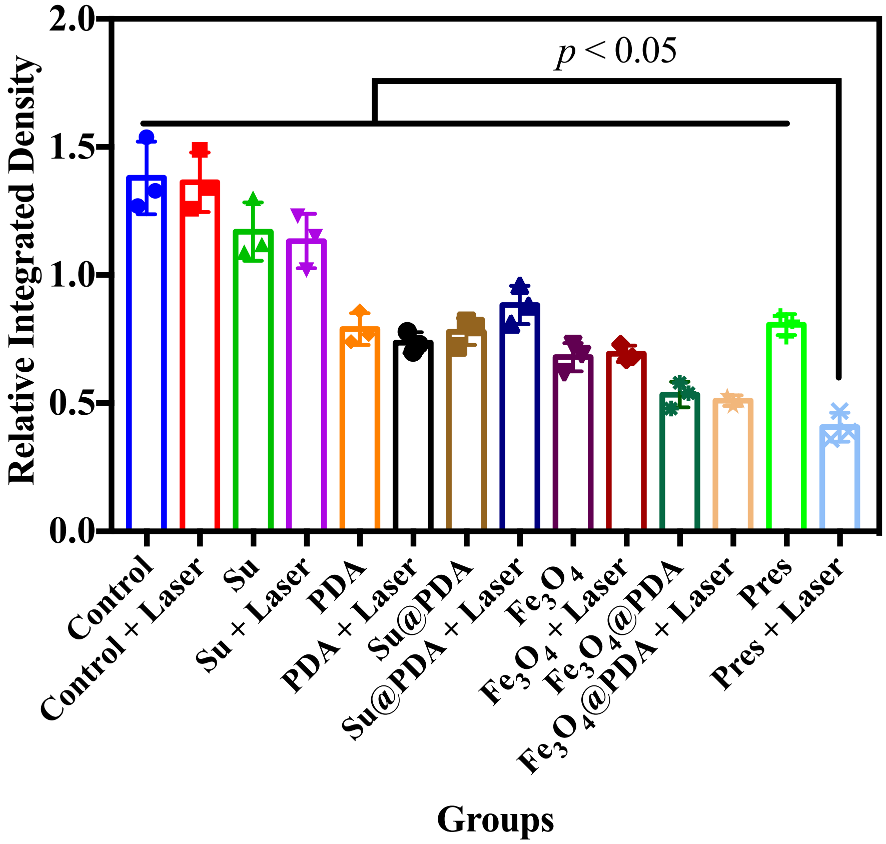

Supplementary Fig. 22. Statistical analysis of relative integrated density calculated via ratios of the gray value of PD-L1 to that of GAPDH in each group in Supplementary Fig. 21. n=3.

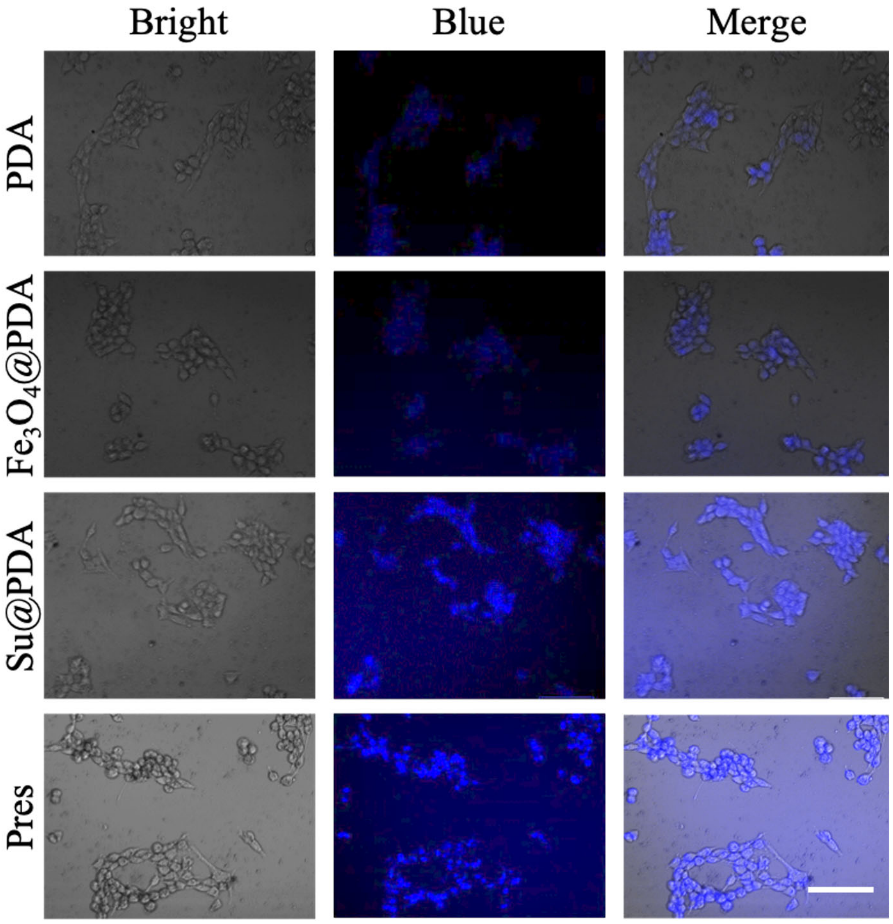

Supplementary Fig. 23. Cellular uptake images of 4T1 cells with different treatments.

scale bar, 100 μm.

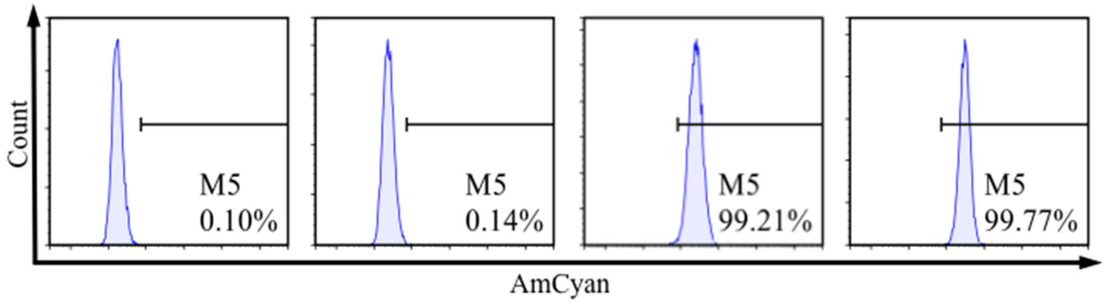

Supplementary Fig. 24. Cellular uptake on 4T1 cells with different treatments by FCM.

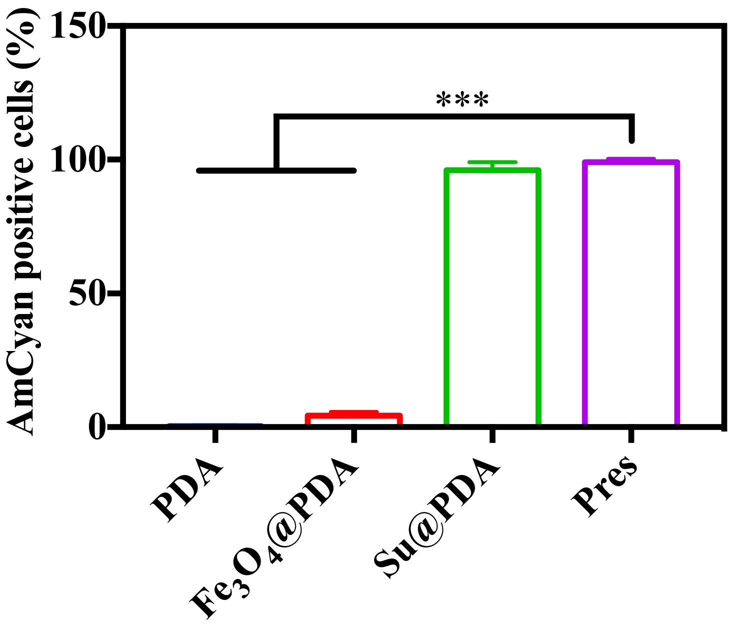

Supplementary Fig. 25. Quantitative analysis of cellular uptake on 4T1 cells with different treatments. *** $p < 0.001$.

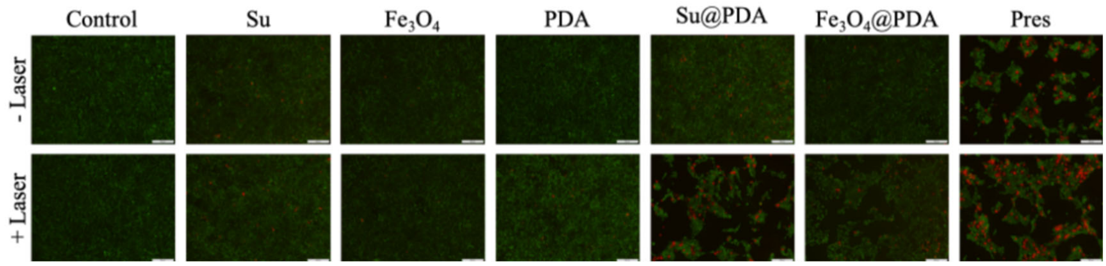

Supplementary Fig. 26. Live-dead staining assay of 4T1 cells with different treatments detected by fluorescence microscope. Scale bar, 200 μm.

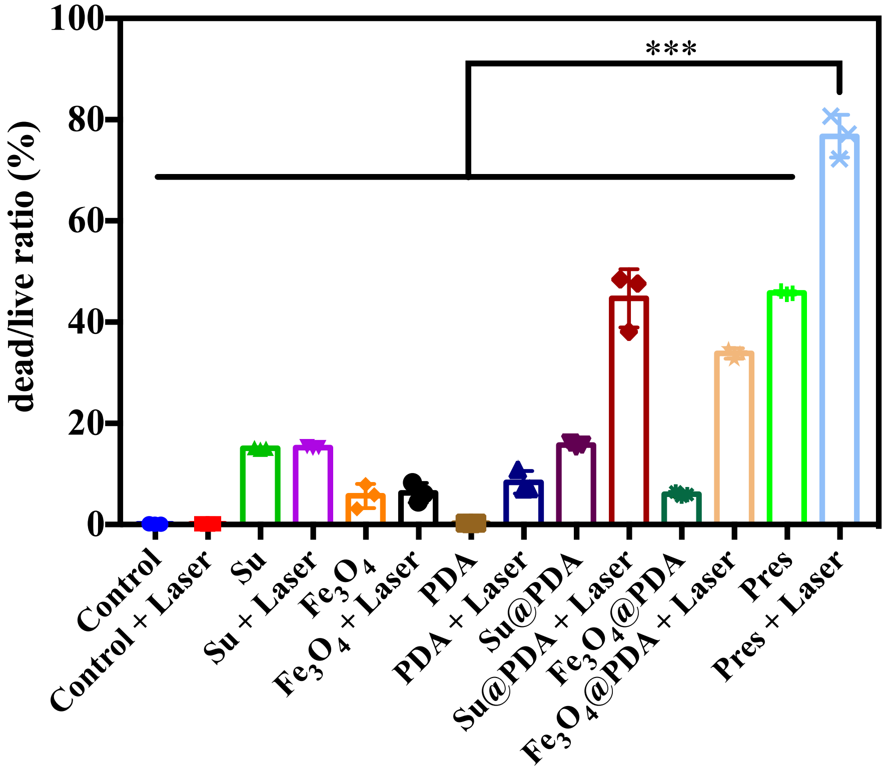

Supplementary Fig. 27. Live/dead ratios of 4T1 cells with different treatments detected by FCM. n=3, *** $p < 0.001$.

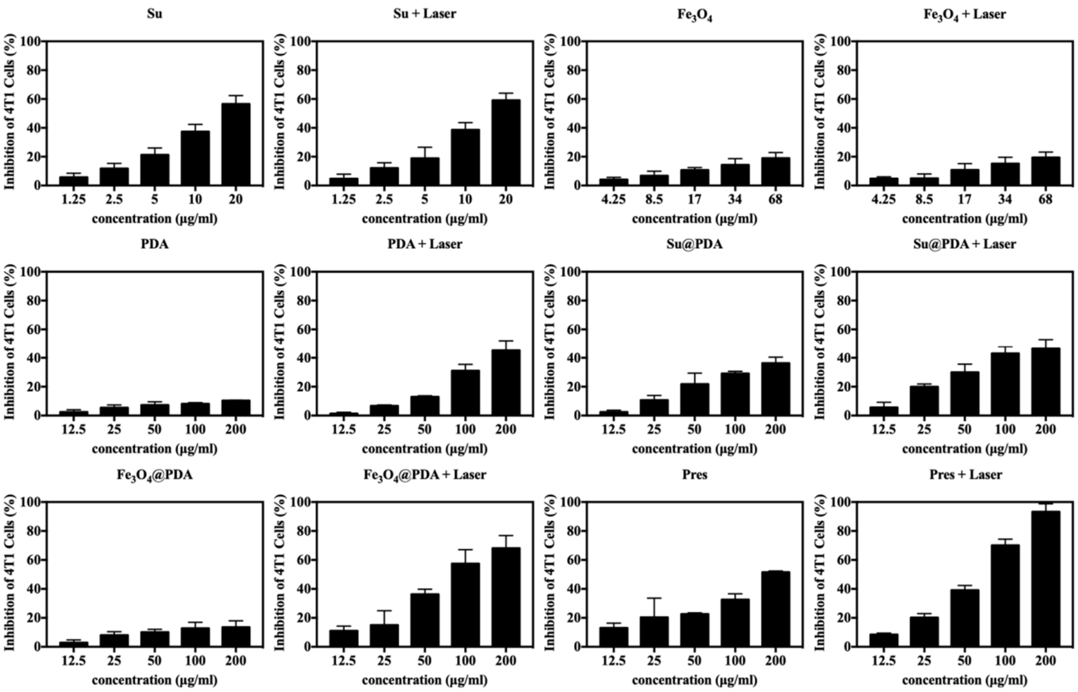

Supplementary Fig. 28. MTT assay on 4T1 cells with different treatments (n=3).

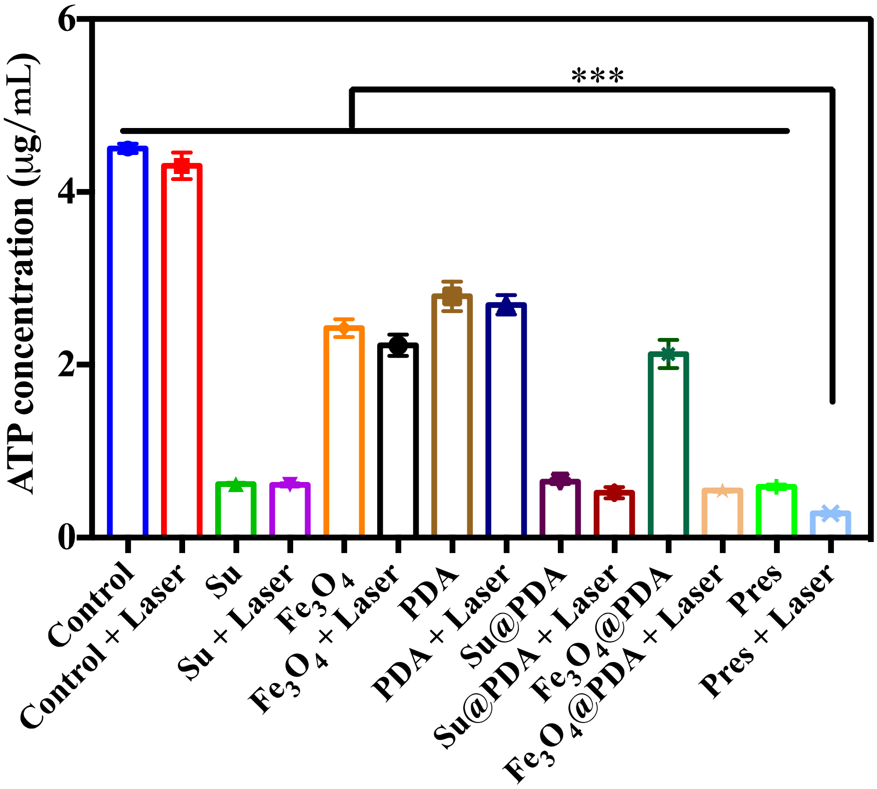

Supplementary Fig. 29. ATP concentrations in 4T1 cells with different treatments.

*** $p < 0.001$.

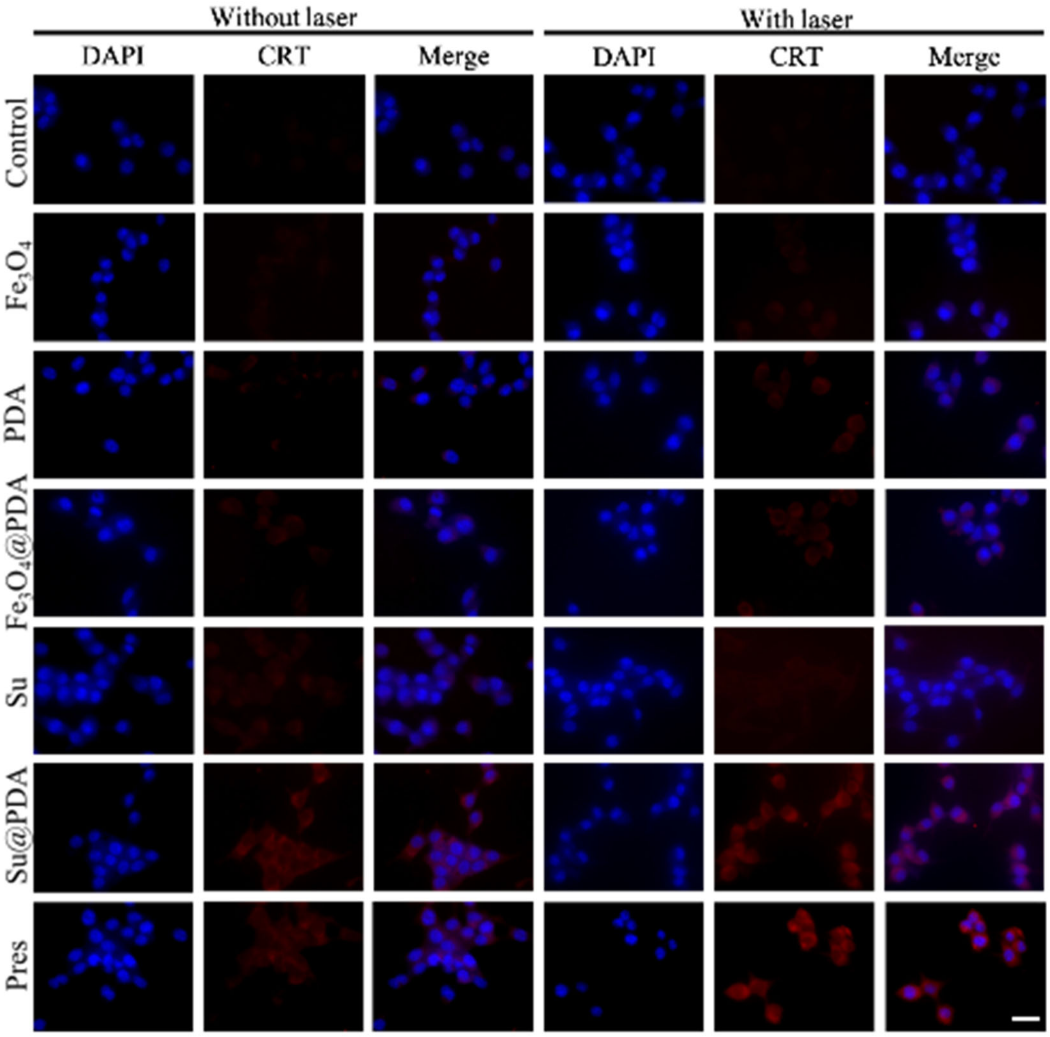

Supplementary Fig. 30. CLSM images of CRT exposure in 4T1 cells with different treatments. Cell nucleuses were stained with DAPI (blue). Scale bar, 100 μm.

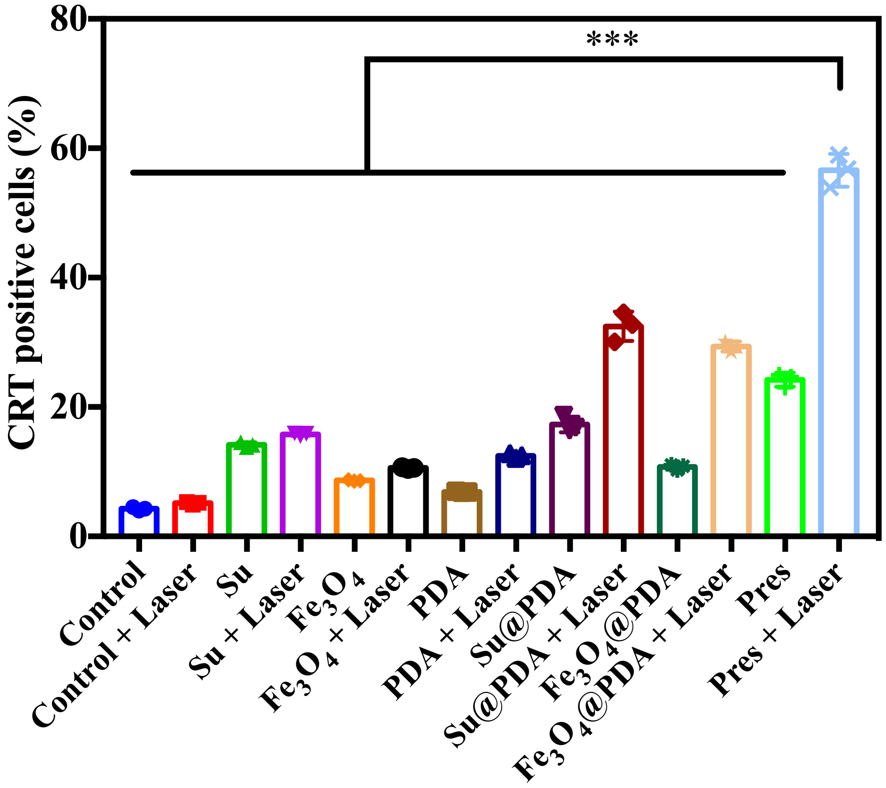

Supplementary Fig. 31. FCM analysis of CRT levels in 4T1 cells with different treatments. *** $p < 0.001$.

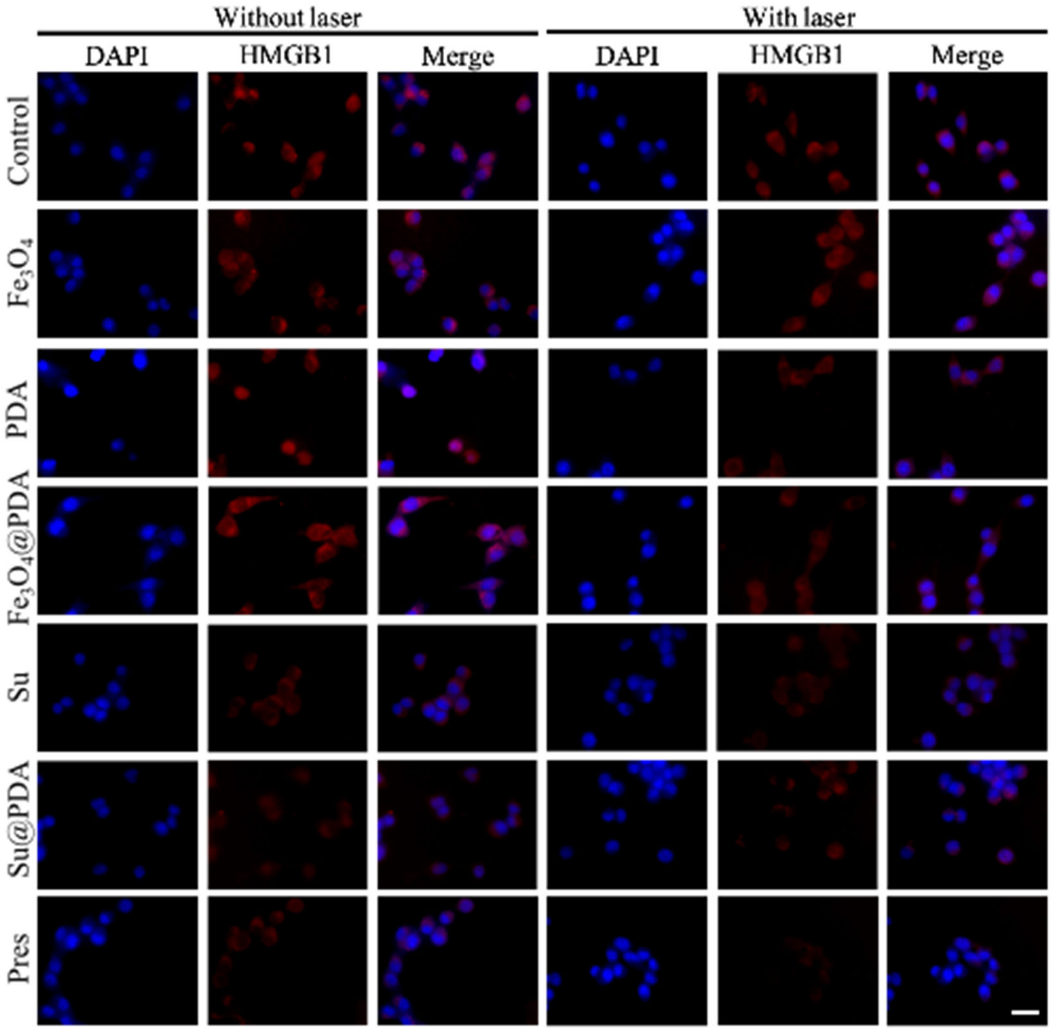

Supplementary Fig. 32. CLSM images of HMGB1 residue in 4T1 cells with different treatments. Cell nucleuses were stained with DAPI (blue). Scale bar, 100 μm.

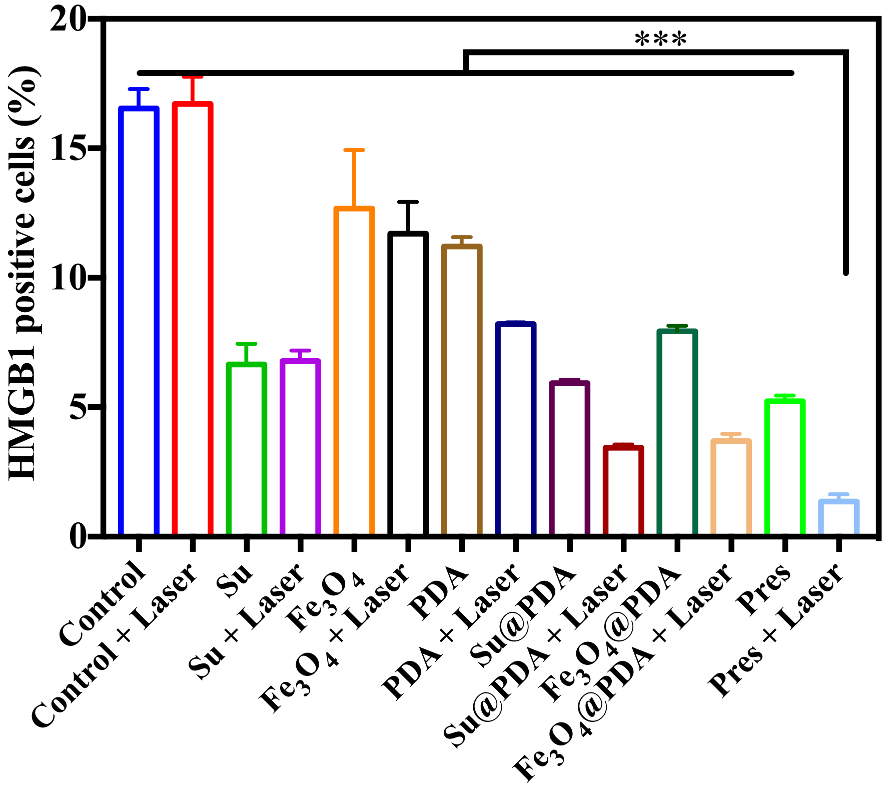

Supplementary Fig. 33. FCM analysis of HMGB1 levels in 4T1 cells with different treatments. *** $p < 0.001$.

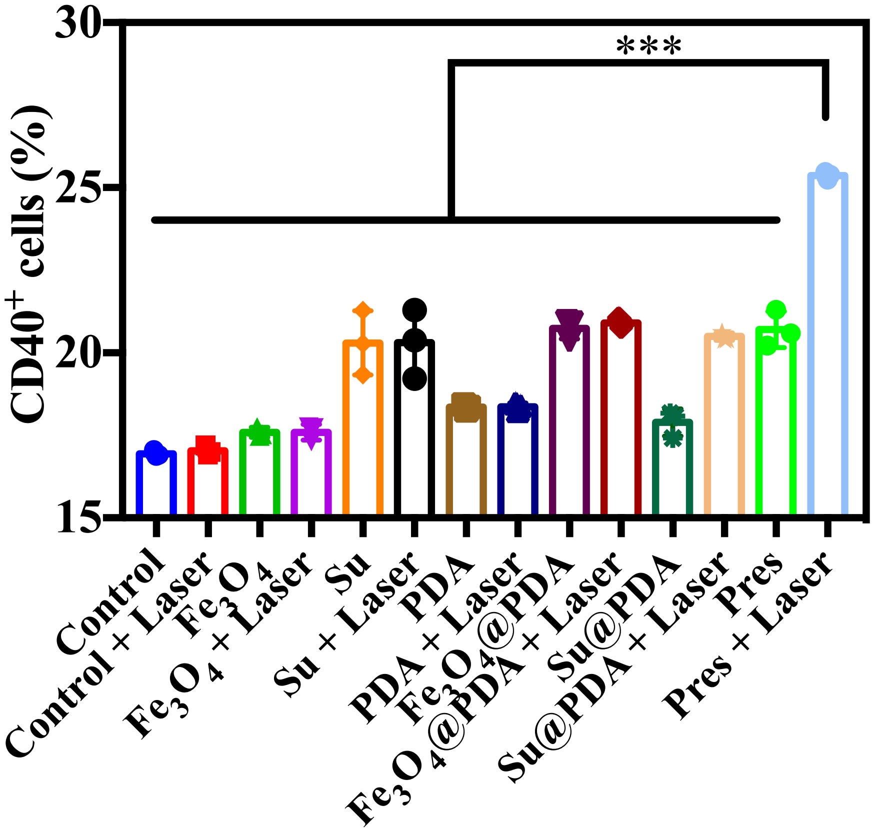

Supplementary Fig. 34. CD11c+CD40+ BMDCs induced by 4T1 cells with different pre-treatments detected by FCM. n=3, *** $p < 0.001$.

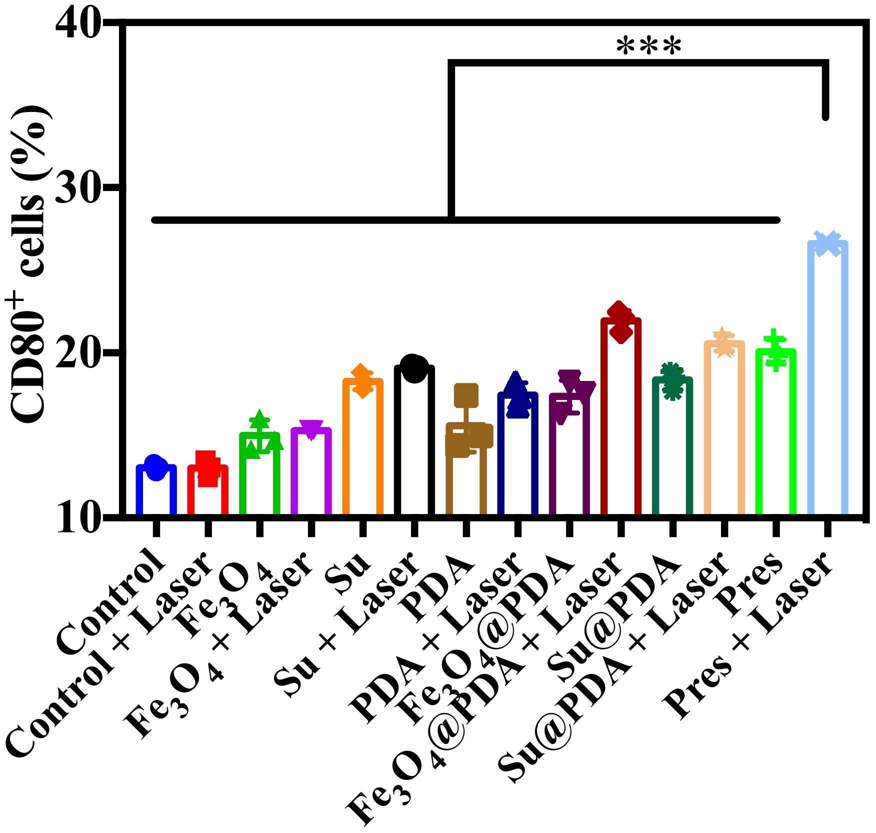

Supplementary Fig. 35. CD11c+CD80+ BMDCs induced by 4T1 cells with different pre-treatments detected by FCM. n=3, *** $p < 0.001$.

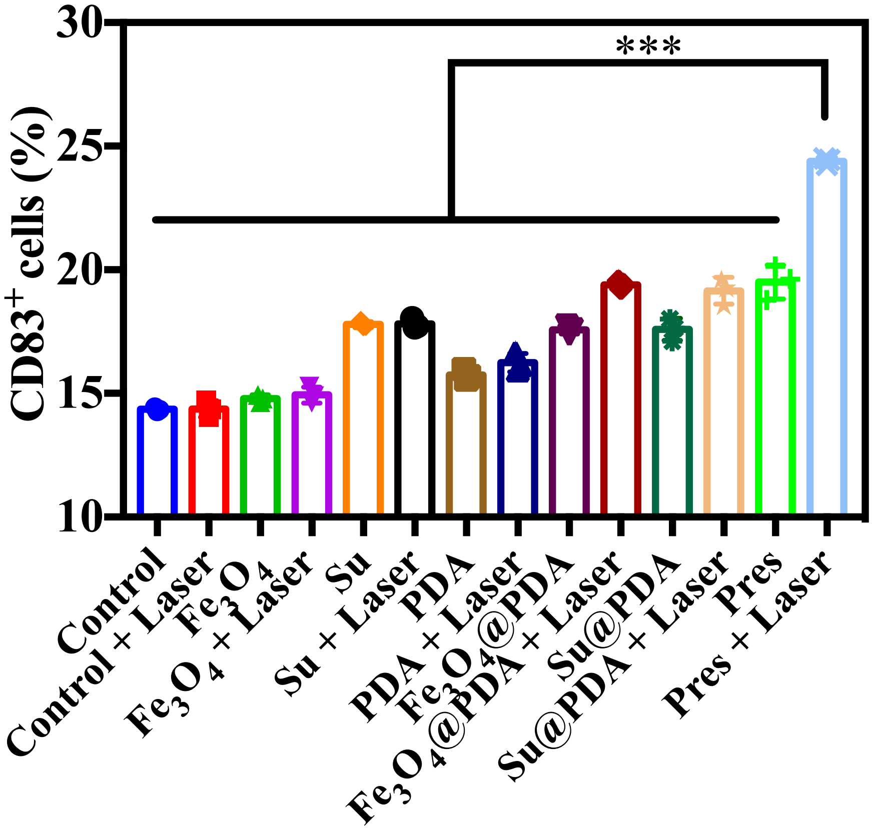

Supplementary Fig. 36. CD11c+CD83+ BMDCs induced by 4T1 cells with different pre-treatments detected by FCM. n=3, *** $p < 0.001$.

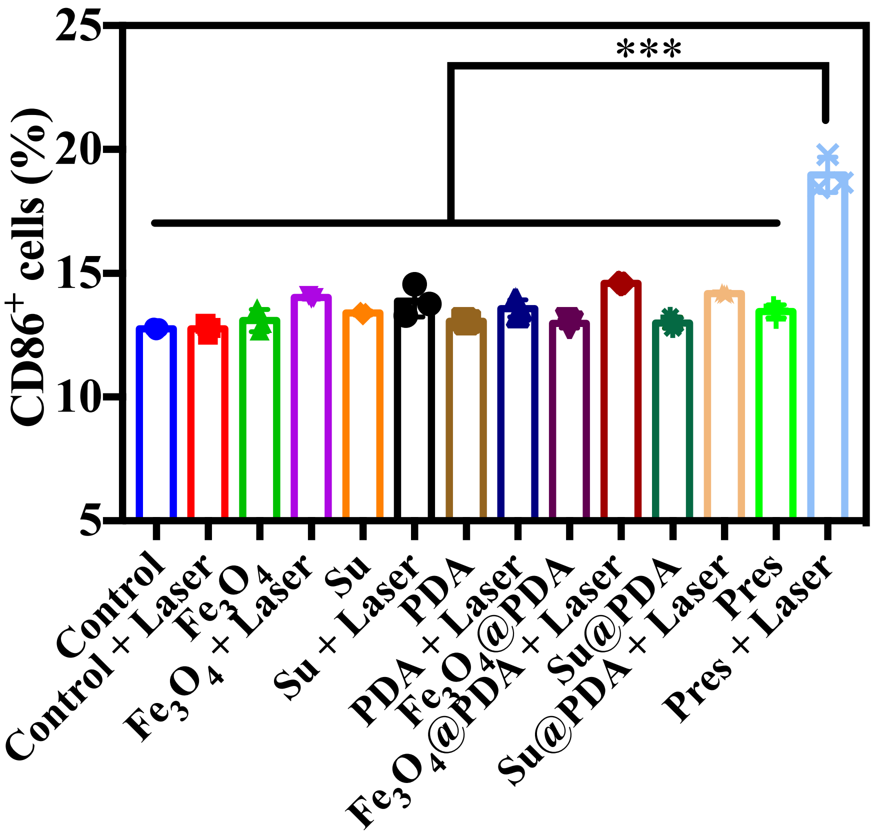

Supplementary Fig. 37. CD11c+CD86+ BMDCs induced by 4T1 cells with different pre-treatments detected by FCM. n=3, *** $p < 0.001$.

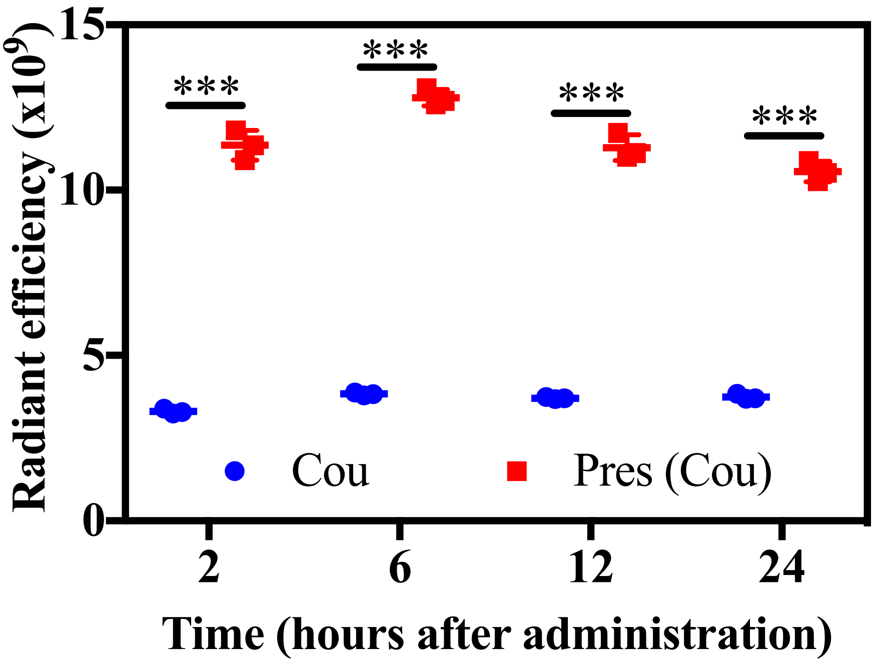

Supplementary Fig. 38. Quantitative analysis of Pres (Cou) biodistribution in 4T1 tumor bearing mice at different time intervals (n=3). *** $p < 0.001$.

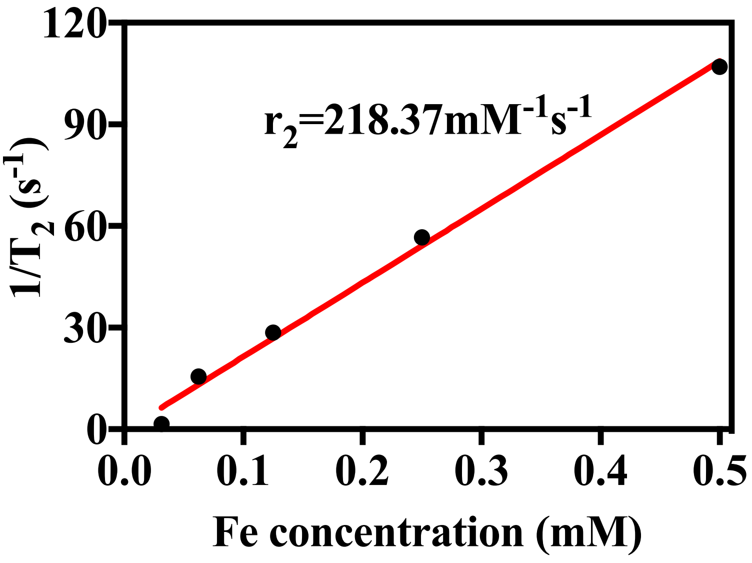

Supplementary Fig. 39. T2 relaxation rates (r2) of Pres according to the left image in

Fig. 4d.

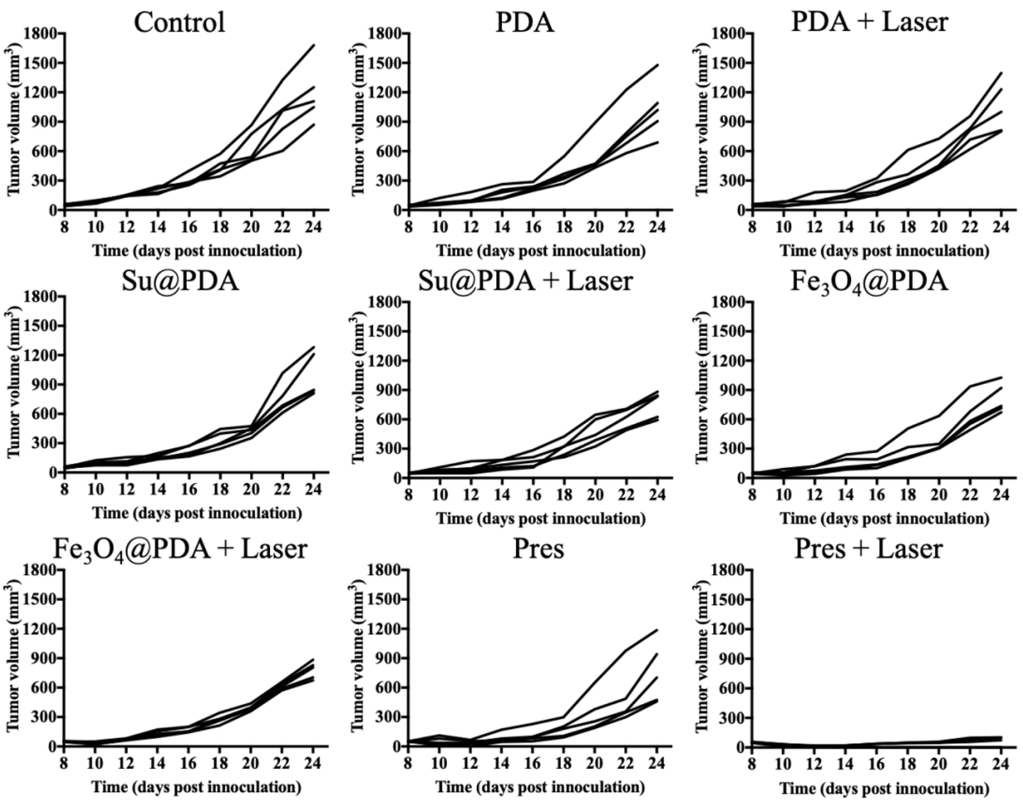

Supplementary Fig. 40. Tumor growth curves of individual tumor bearing mice with different treatments during the observation period in unilateral subcutaneous 4T1 tumor models. n=5.

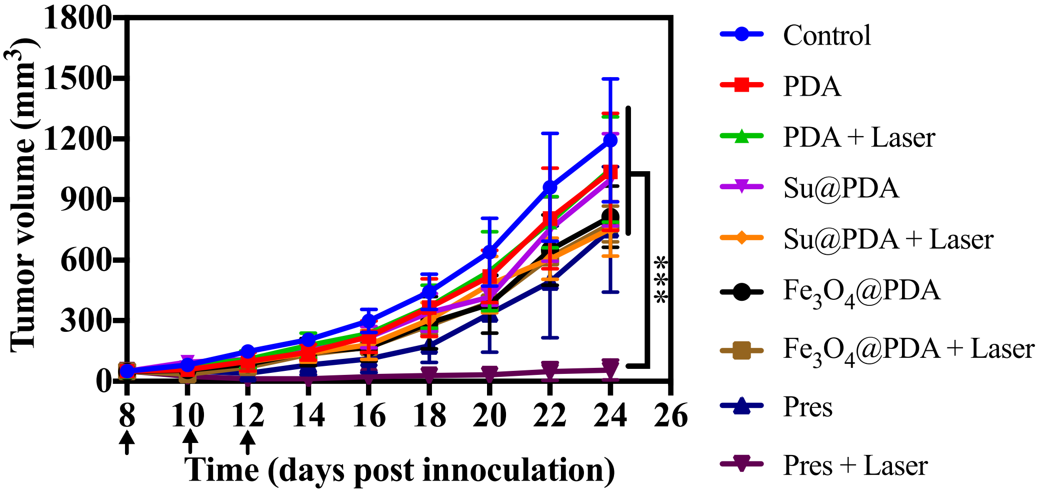

Supplementary Fig. 41. Tumor growth curves of subcutaneous 4T1 tumors (n=5). The arrows indicated treatments. *** $p < 0.001$.

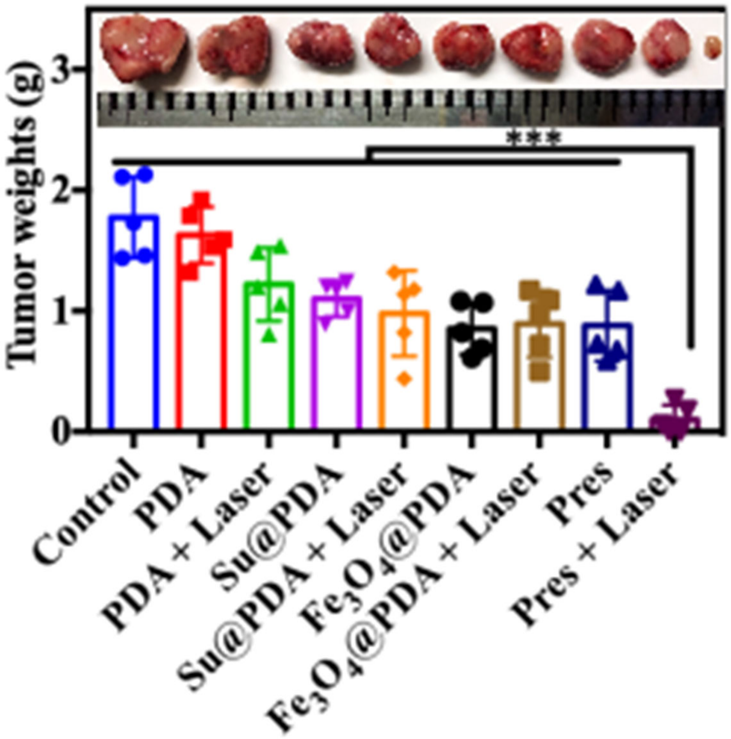

Supplementary Fig. 42. Weights of ex vivo tumors (n=5). Representative tumor images were inserted. The group of each tumor in the inserted image correlated with the group below from left to right. *** $p < 0.001$.

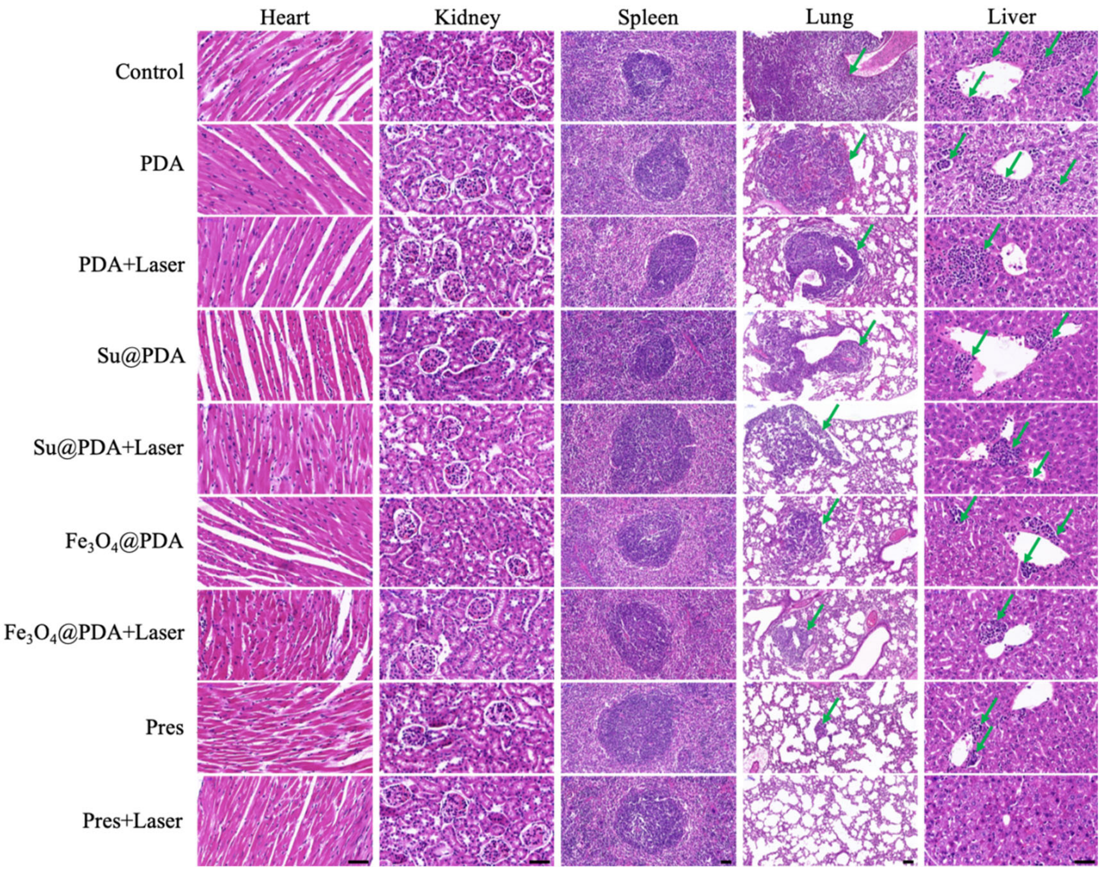

Supplementary Fig. 43. H&E staining of ex vivo heart, kidney, spleen, lung and liver with different treatments. The green arrows pointed at the metastases in lungs and livers. Scale bar, 50 μm.

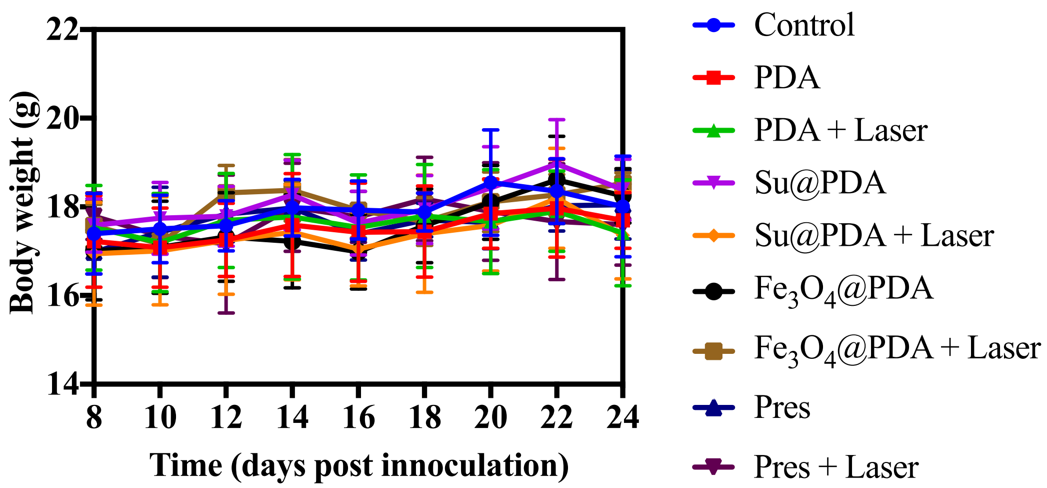

Supplementary Fig. 44. Body weight changes of mice with different treatments during the treatment period (n=5).

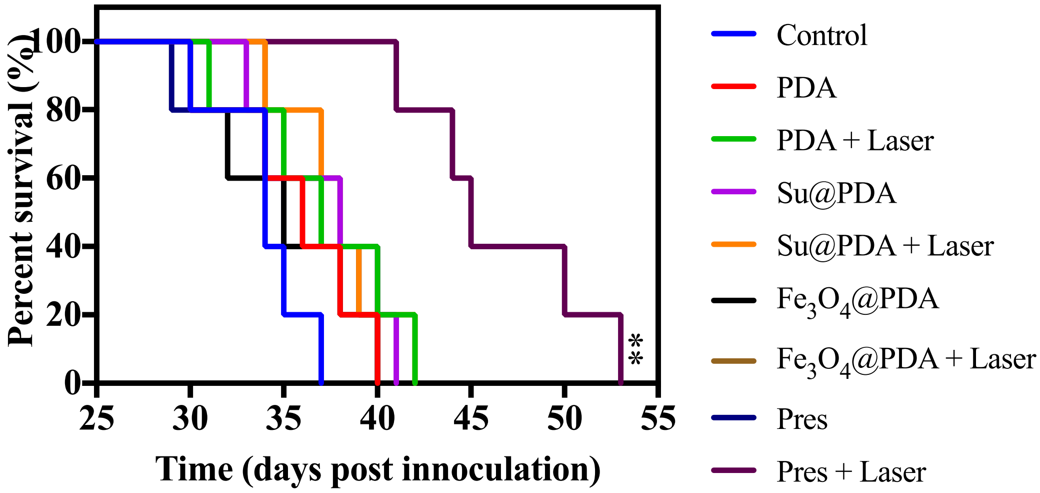

Supplementary Fig. 45. Survival curve of tumor-bearing mice with different treatments. n=5, ** $p < 0.01$.

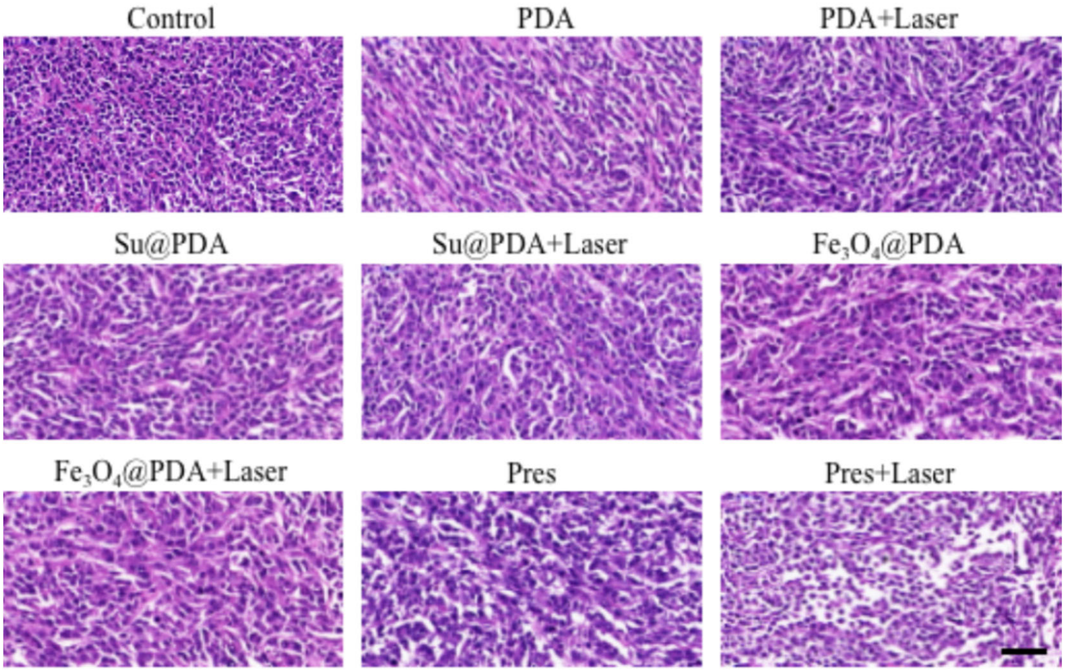

Supplementary Fig. 46. H&E staining of *ex vivo* tumors. Scale bar, 50 µm.

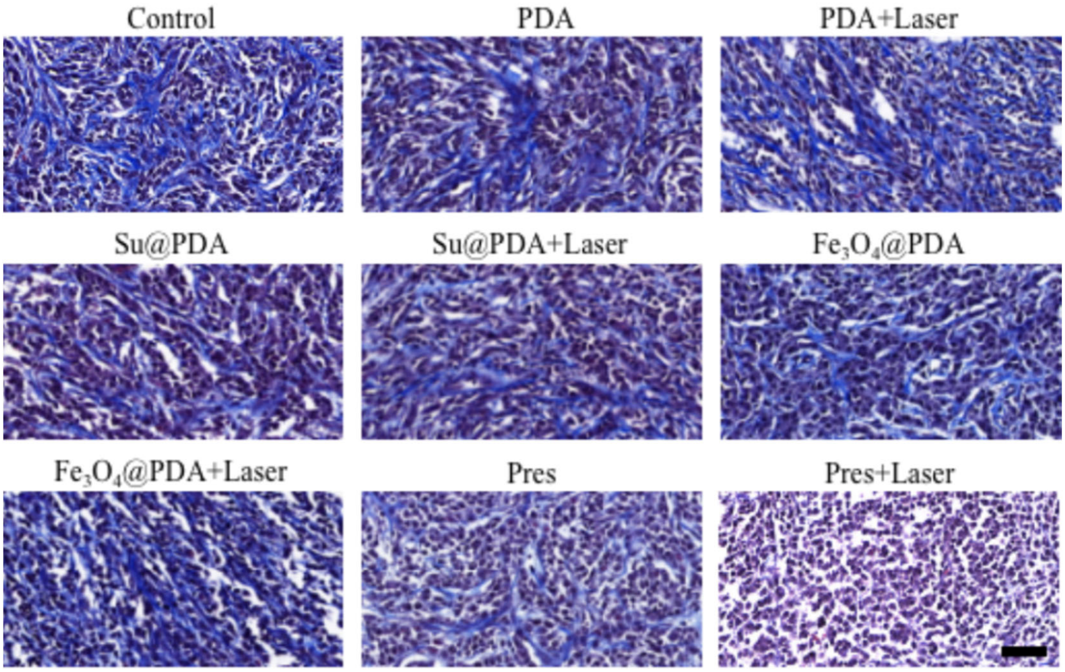

Supplementary Fig. 47. Masson staining of ex vivo tumors. Scale bar, 50 µm.

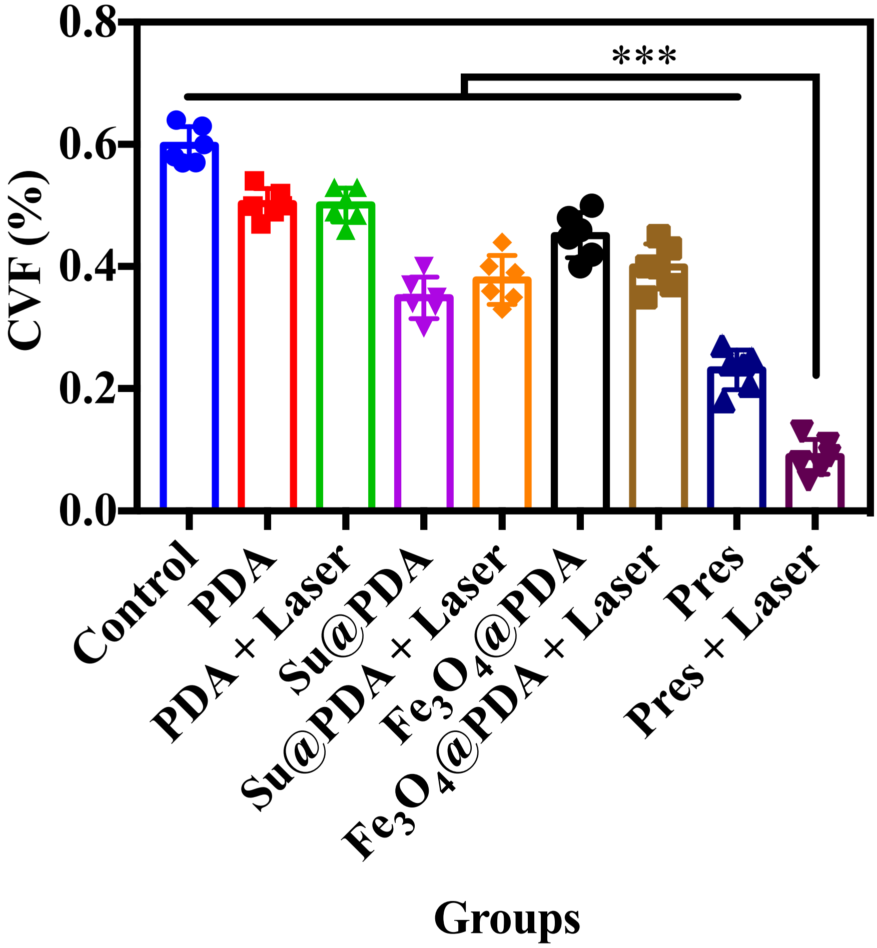

Supplementary Fig. 48. CVF value of masson staining of ex vivo tumors from mice with different treatments, n=6, *** indicates p < 0.001.

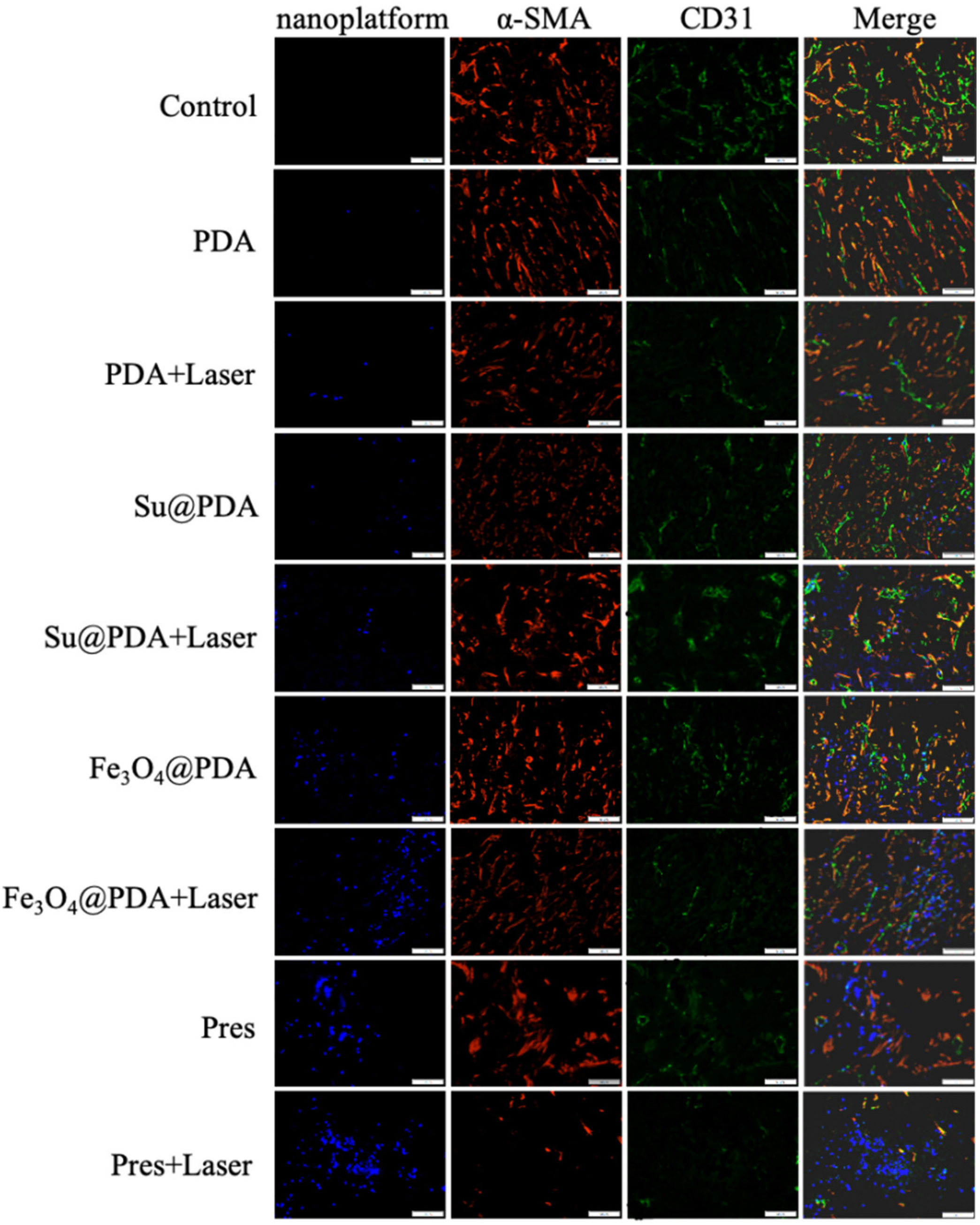

Supplementary Fig. 49. Immunofluorescence staining of α-SMA and CD31 ex vivo tumors from mice with different treatments, scale bar, 50 μm.

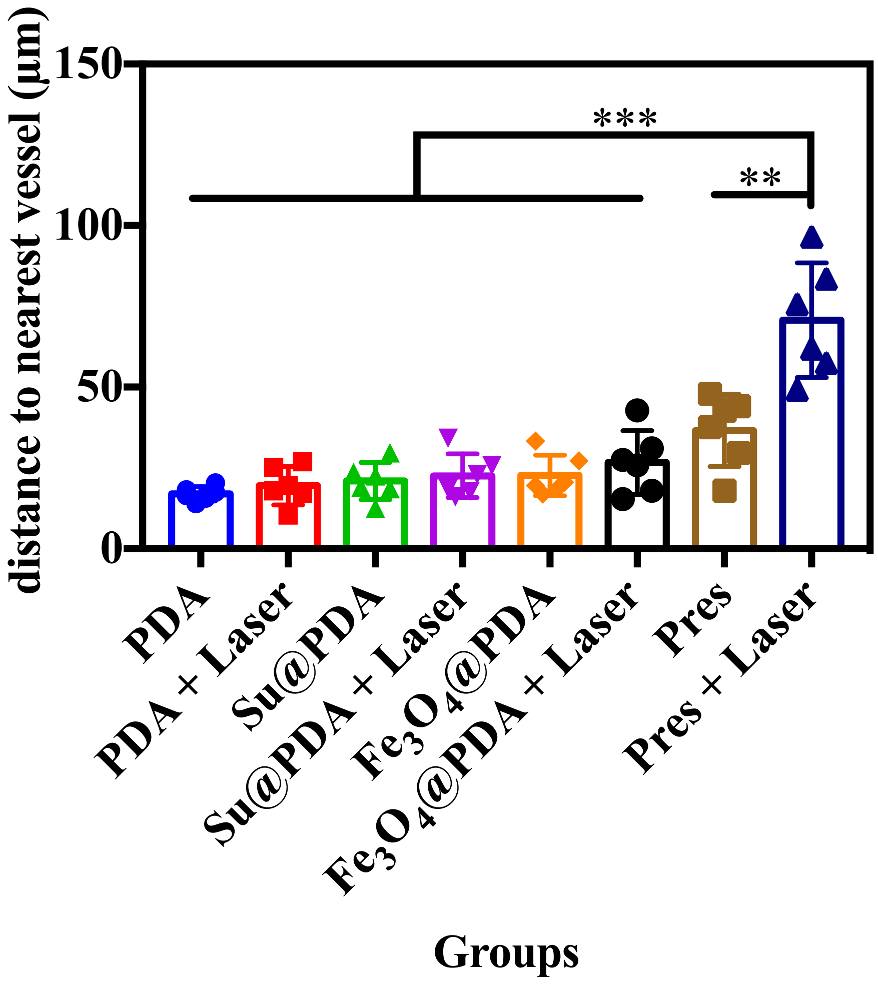

Supplementary Fig. 50. Distances of nanoplatforms to the nearest vessel, ** indicates *p* < 0.01, *** indicates p < 0.001.

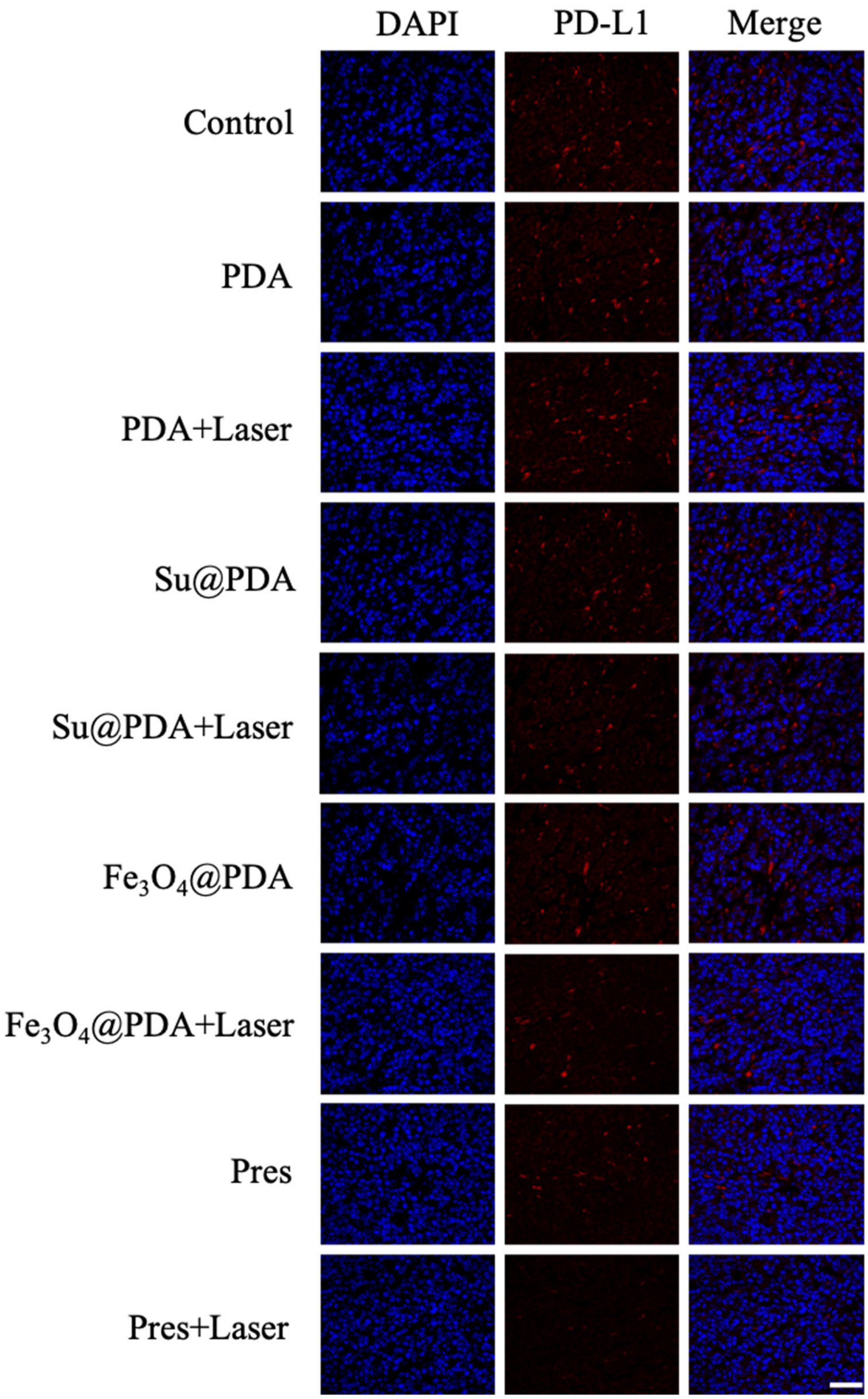

Supplementary Fig. 51. Immunofluorescence staining of PD-L1 in ex vivo tumors from mice with different treatments, scale bar, 100 μm.

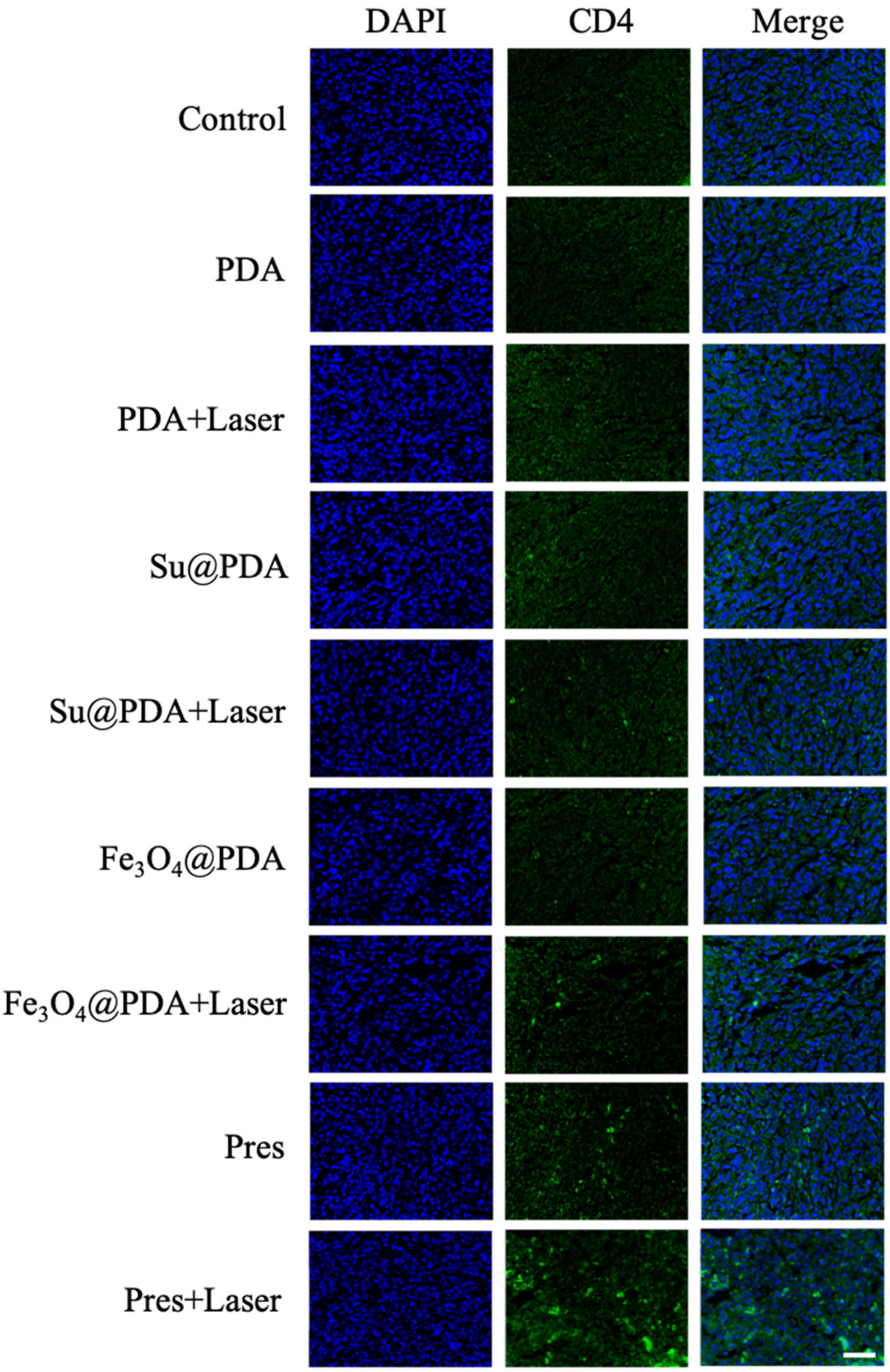

Supplementary Fig. 52. Immunofluorescence staining of CD4 in ex vivo tumors from mice with different treatments, scale bar, 100 μm.

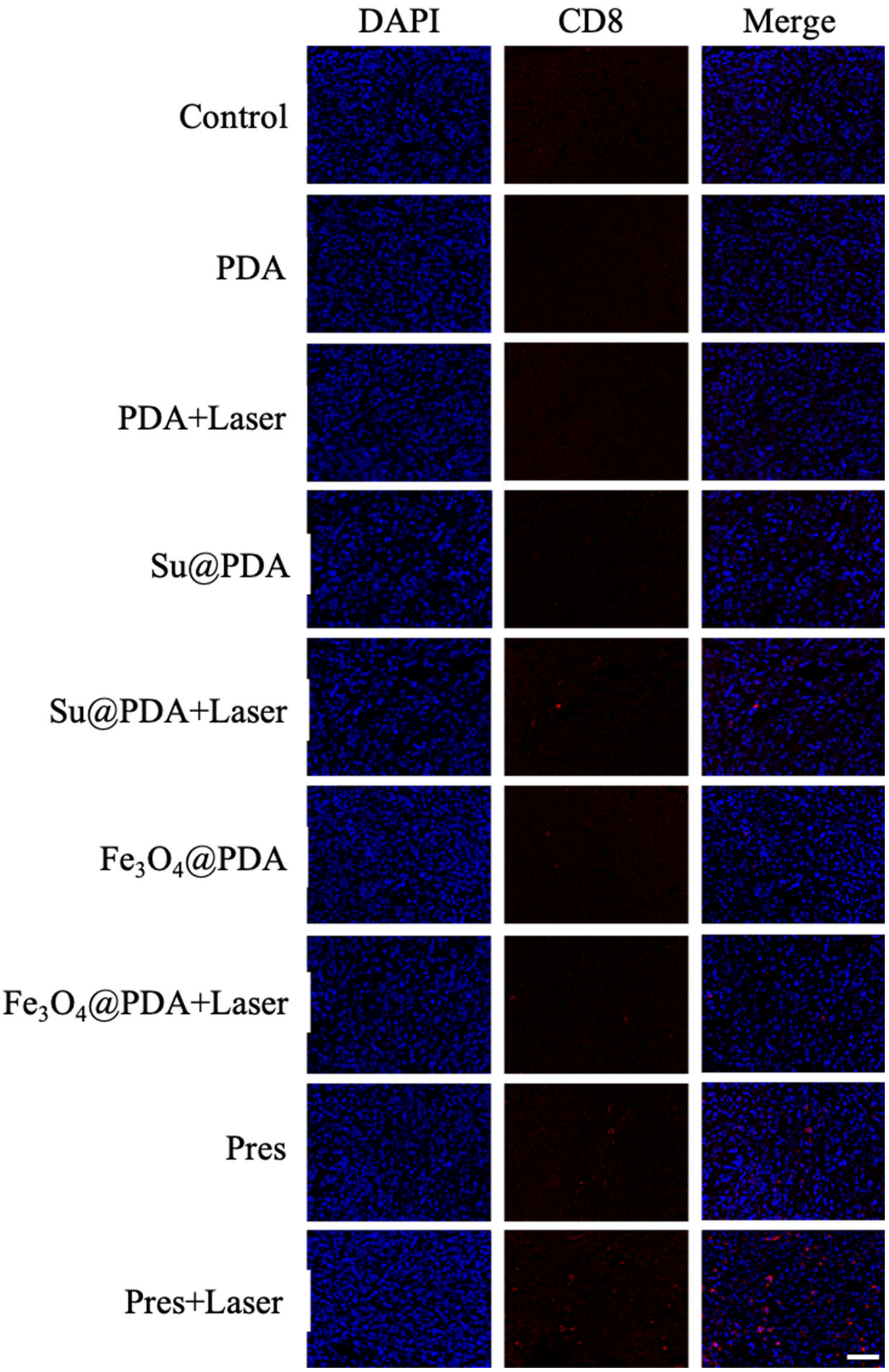

Supplementary Fig. 53. Immunofluorescence staining of CD8 in ex vivo tumors from mice with different treatments, scale bar, 100 μm.

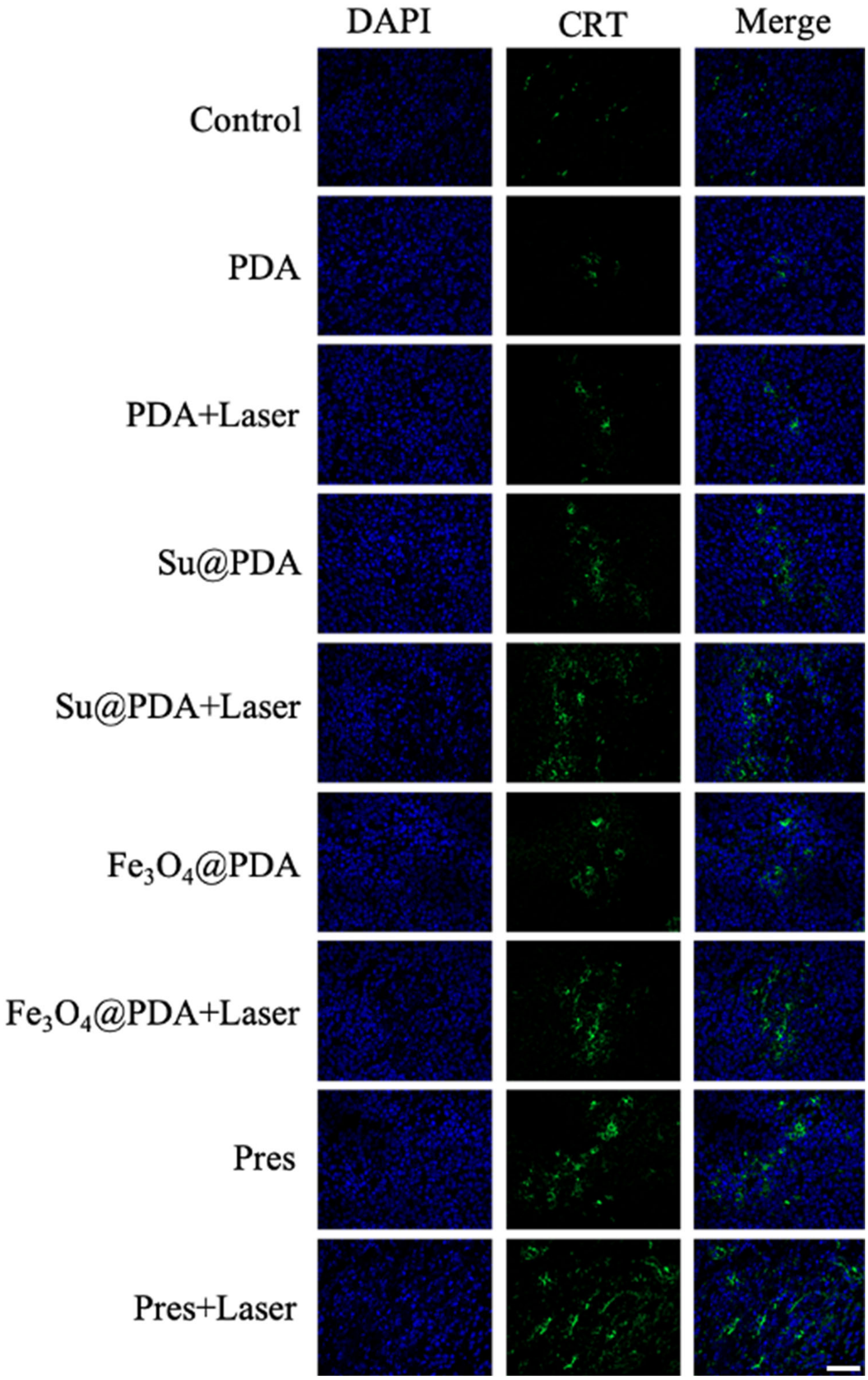

Supplementary Fig. 54. Immunofluorescence staining of CRT in ex vivo tumors from mice with different treatments, scale bar, 100 μm.

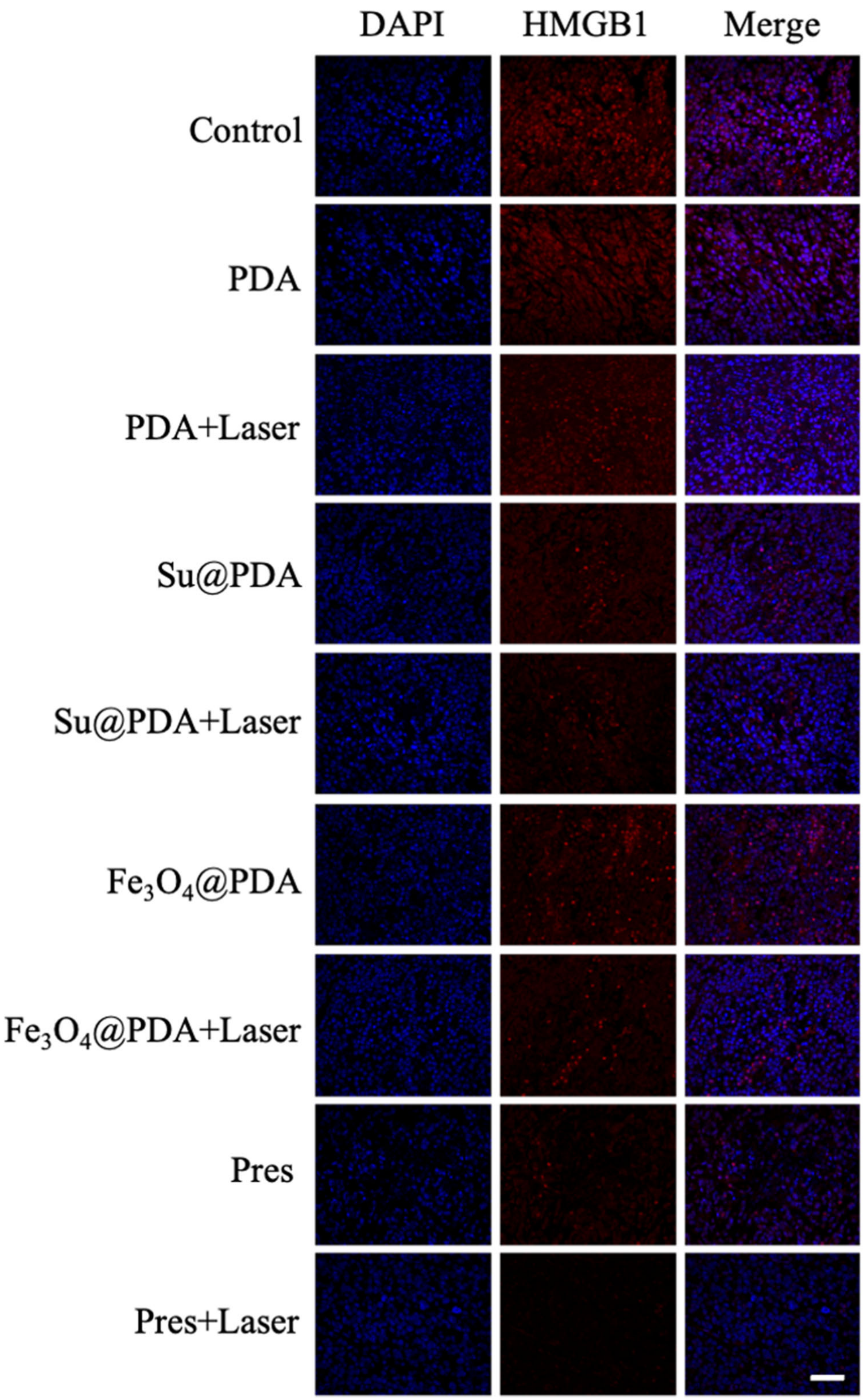

Supplementary Fig. 55. Immunofluorescence staining of HMGB1 in ex vivo tumors from mice with different treatments, scale bar, 100 μm.

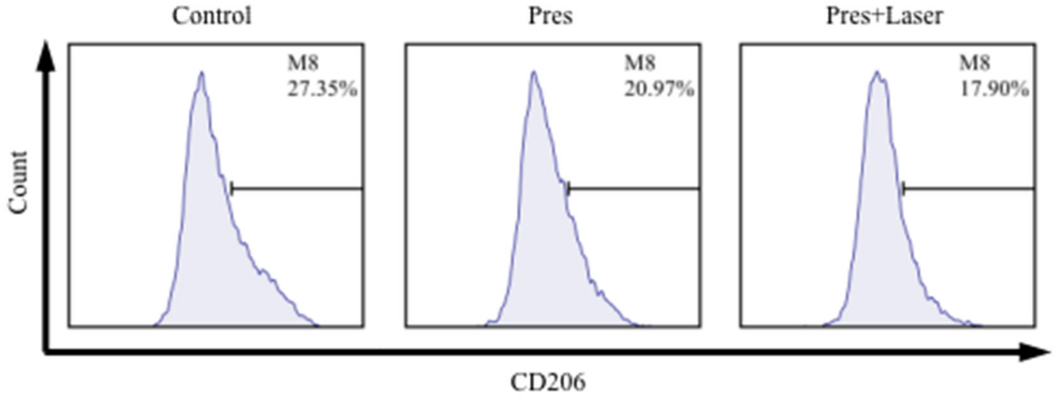

Supplementary Fig. 56. FCM analysis of M2 TAM populations according to $CD11b^{+}F4/80^{+}CD206^{+}$.

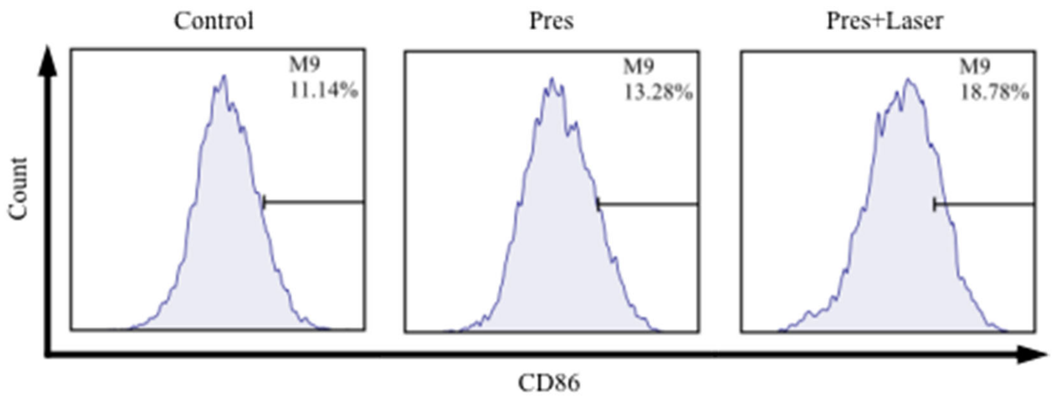

Supplementary Fig. 57. FCM analysis of M1 TAM populations according to $CD11b^{+}F4/80^{+}CD86^{+}$.

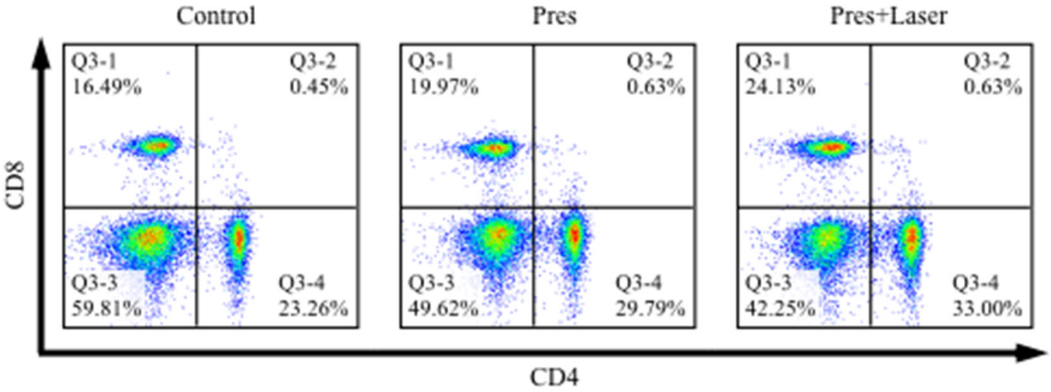

Supplementary Fig. 58. FCM analysis of $CD3^{+}CD4^{+}$ and $CD3^{+}CD8^{+}$ T cells in lymph nodes.

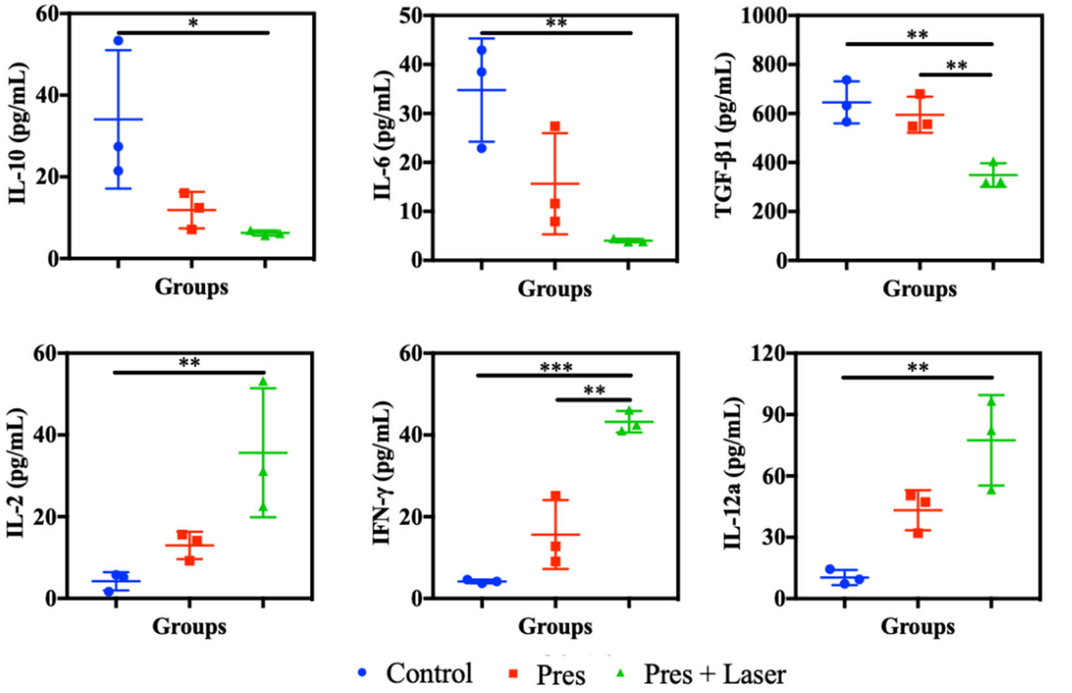

Supplementary Fig. 59. IL-10, IL-6, TGF-β (Th2 cytokines) and IL-2, IFN-γ, IL-12a (Th1 cytokines) levels in serums of different groups. Representative groups of different colors were denoted in the bottom. * $p < 0.05$, ** $p < 0.01$, *** $p < 0.001$.

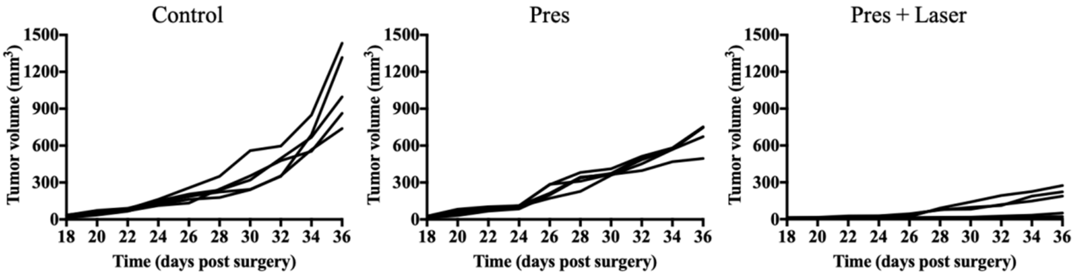

Supplementary Fig. 60. Distant tumor volume changes of individual tumor bearing mice with PBS, Pres with or without laser irradiation (808 nm, 5 min, 1.0 W cm^{-2}) treatments during the observation period in distal 4T1 models. n=5.

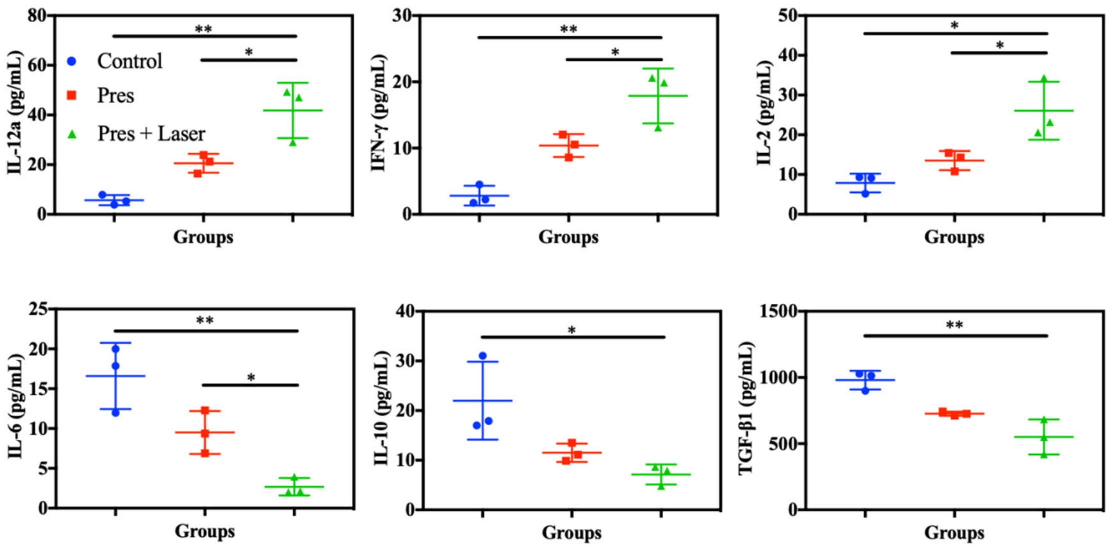

Supplementary Fig. 61. IL-10, IL-6, TGF-β (Th2 cytokines) and IL-2, IFN-γ, IL-12a (Th1 cytokines) levels in serums of mice with different pre-treatments in immune memory experiments (n=3). * $p < 0.05$, ** $p < 0.01$.

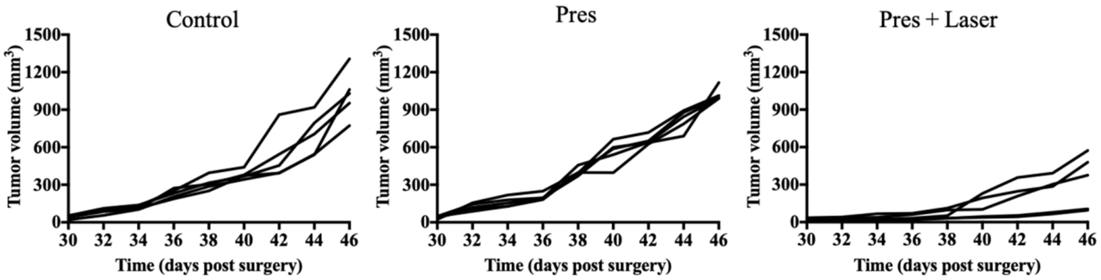

Supplementary Fig. 62. Second tumor volume changes of individual tumor bearing mice with PBS, Pres with or without laser irradiation (808 nm, 5 min, 1.0 W cm^{-2}) treatments during the observation period in rechallenge 4T1 models. n=5.
